# Supplementary material for: Ultra-wideband-responsive photon conversion through co-sensitization in lanthanide nanocrystals
Source: Nat Commun. 2023 Feb 14;14:827. doi: 10.1038/s41467-023-36510-3 (PMC9929054; doi:10.1038/s41467-023-36510-3)
Supplement: Supplementary file 1 — Supplementary Information [file 41467_2023_36510_MOESM1_ESM.pdf]

Supplementary Information

Ultra-wideband-responsive photon conversion through co-sensitization in lanthanide nanocrystals

Zhao Jiang[1], Liangrui He[1], Zhiwen Yang[1], Huibin Qiu[2], Xiaoyuan Chen[3], Xujiang Yu[1*] and Wanwan Li[1*]

[1] State Key Lab of Metal Matrix Composites
School of Materials Science and Engineering
Zhangjiang Institute for Advanced Study
Shanghai Jiao Tong University
800 Dongchuan Road, Shanghai 200240, P. R. China

[2] State Key Laboratory of Metal Matrix Composites
Frontiers Science Centre for Transformative Molecules
School of Chemistry and Chemical Engineering
Shanghai Jiao Tong University
Shanghai 200240, P. R. China

[3] Yong Loo Lin School of Medicine and Faculty of Engineering
National University of Singapore
Singapore, 117597, Singapore

Table of Contents

Supplementary Figures

Supplementary Fig. 1 3
Supplementary Fig. 2 3
Supplementary Fig. 3 4
Supplementary Fig. 4 4
Supplementary Fig. 5 5
Supplementary Fig. 6 5
Supplementary Fig. 7 6
Supplementary Fig. 8 7
Supplementary Fig. 9 8
Supplementary Fig. 10 9
Supplementary Fig. 11 10
Supplementary Fig. 12 11
Supplementary Fig. 13 12
Supplementary Fig. 14 13
Supplementary Fig. 15 13
Supplementary Fig. 16 14
Supplementary Fig. 17 15
Supplementary Fig. 18 16
Supplementary Fig. 19 16
Supplementary Fig. 20 17
Supplementary Fig. 21 17
Supplementary Fig. 22 18
Supplementary Fig. 23 18
Supplementary Fig. 24 19
Supplementary Fig. 25 19
Supplementary Fig. 26 20
Supplementary Fig. 27 21
Supplementary Fig. 28 22
Supplementary Fig. 29 23
Supplementary Fig. 30 23
Supplementary Fig. 31 24
Supplementary Fig. 32 25
Supplementary Fig. 33 25
Supplementary Fig. 34 26
Supplementary Fig. 35 26
Supplementary Fig. 36 26
Supplementary Fig. 37 27
Supplementary Fig. 38 27
Supplementary Fig. 39 27
Supplementary Fig. 40 28
Supplementary Fig. 41 29
Supplementary Fig. 42 31
Supplementary Fig. 43 31
Supplementary Fig. 44 31
Supplementary Fig. 45 31

Supplementary Tables

Supplementary Table 1 10

Supplementary References

Supplementary References 32

Supplementary Figures and Tables

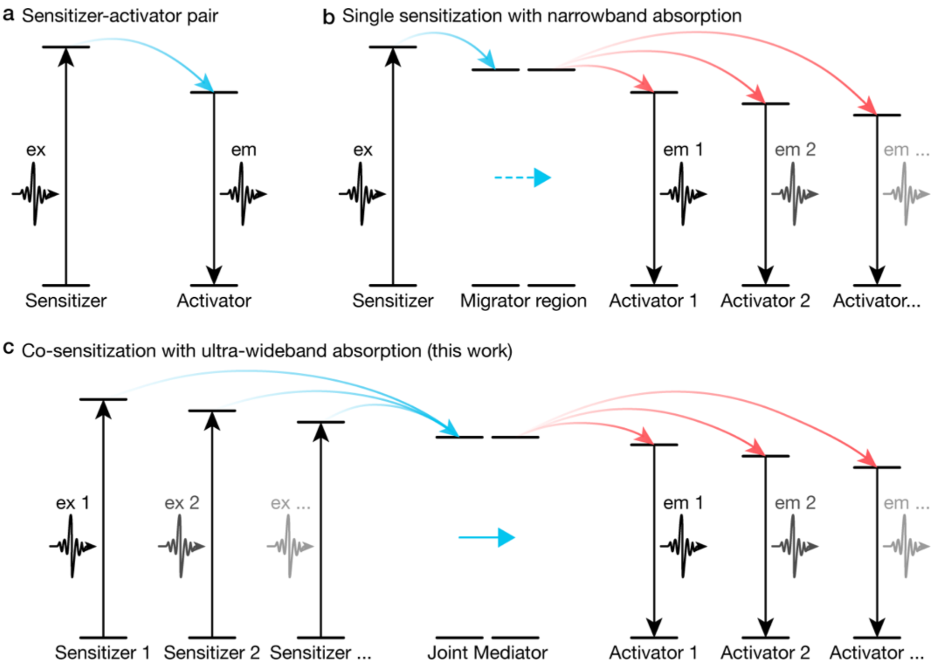

Supplementary Fig. 1 | Schematic illustration and tunning of sensitization strategies. a-c, Illustration of the ET within a sensitizer-activator pair (**a**), a single sensitization structure (**b**), and the proposed co-sensitization structure (**c**).

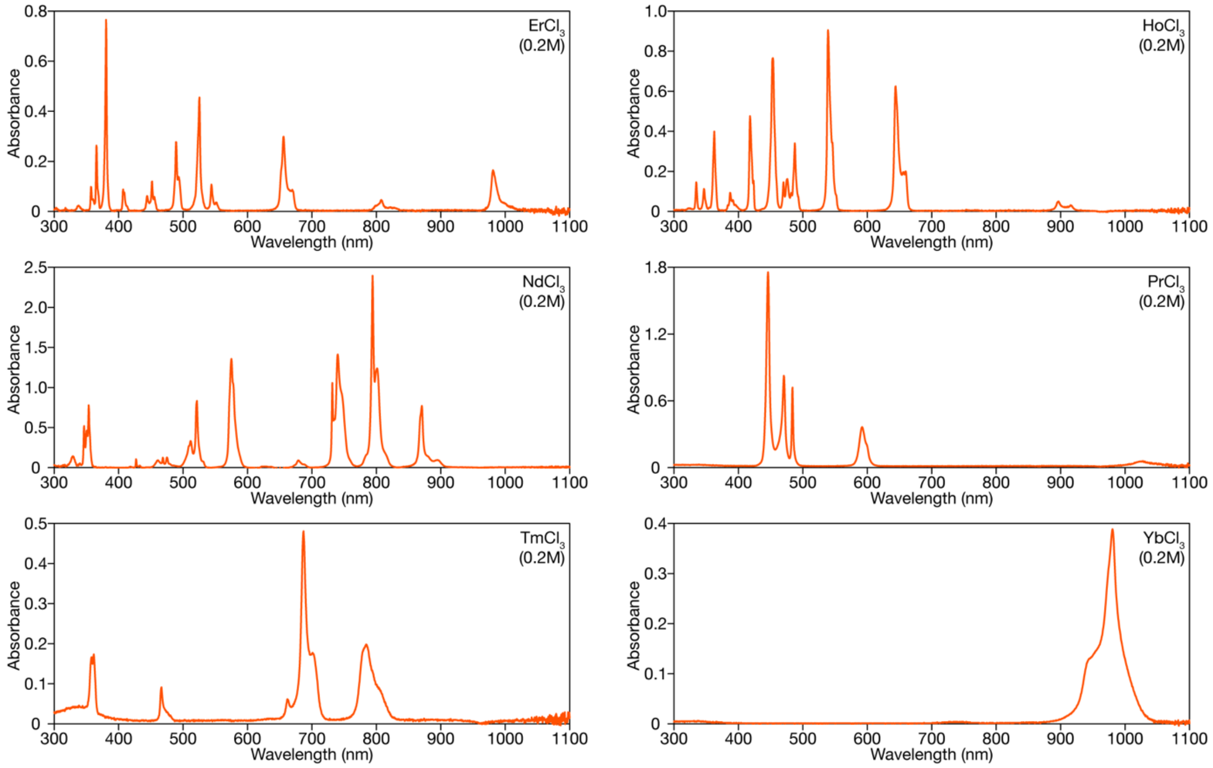

Supplementary Fig. 2 | Absorption spectra of $ErCl_3$, $HoCl_3$, $NdCl_3$, $PrCl_3$, $TmCl_3$, and $YbCl_3$ in aqueous solution (0.2 M). These lanthanide ions all showed multiple responsive bands in a wide wavelength range.

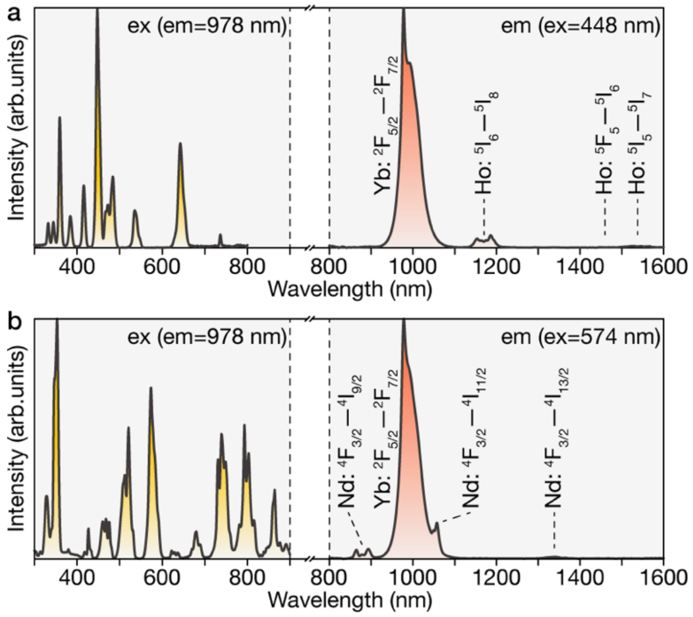

Supplementary Fig. 3 | a, Excitation and emission spectra of $NaHoF_4:Yb^{3+}$. **b**, Excitation and emission spectra of $NaNdF_4:Yb^{3+}$. 448 nm corresponds to the absorption of Ho^{3+}, and 574 nm corresponds to the absorption of Nd^{3+}.

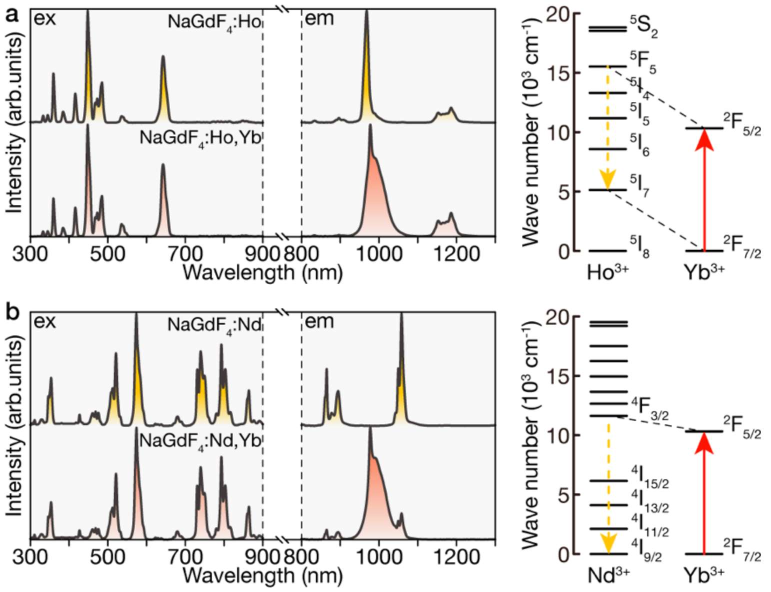

Supplementary Fig. 4 | a, Excitation and emission spectra of $NaGdF_4:Ho^{3+}$ (ex=448 nm, em=968 nm) and $NaGdF_4:Ho^{3+},Yb^{3+}$ (ex=448 nm, em=978 nm), and the ET between Ho^{3+} and Yb^{3+}. **b**, Excitation and emission spectra of $NaGdF_4:Nd^{3+}$ (ex=574 nm, em=865 nm) and $NaGdF_4:Nd^{3+},Yb^{3+}$ (ex=574 nm, em=978 nm), and the ET between Nd^{3+} and Yb^{3+}.

For $NaGdF_4:Ho^{3+},Yb^{3+}$, the excitation spectrum (by monitoring the emission of Yb^{3+} at 978 nm) completely corresponded to the absorption of Ho^{3+}, indicating that there was an interaction between Ho^{3+} and Yb^{3+}, i.e. energy transfer. It was the same case for $NaGdF_4:Nd^{3+},Yb^{3+}$.

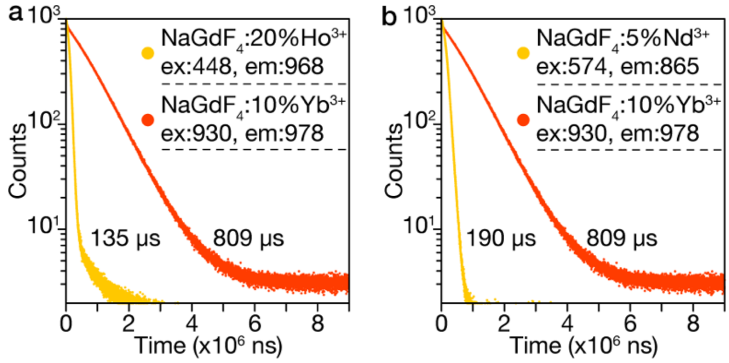

Supplementary Fig. 5 | a, Fluorescence decay curves of $NaGdF_4:Ho^{3+}$ and $NaGdF_4:Yb^{3+}$. **b**, Fluorescence decay curves of $NaGdF_4:Nd^{3+}$ and $NaGdF_4:Yb^{3+}$. The lifetimes of Yb^{3+}, Ho^{3+}, and Nd^{3+} in a $NaGdF_4$ matrix were determined to be 809, 135, and 190 μs, respectively.

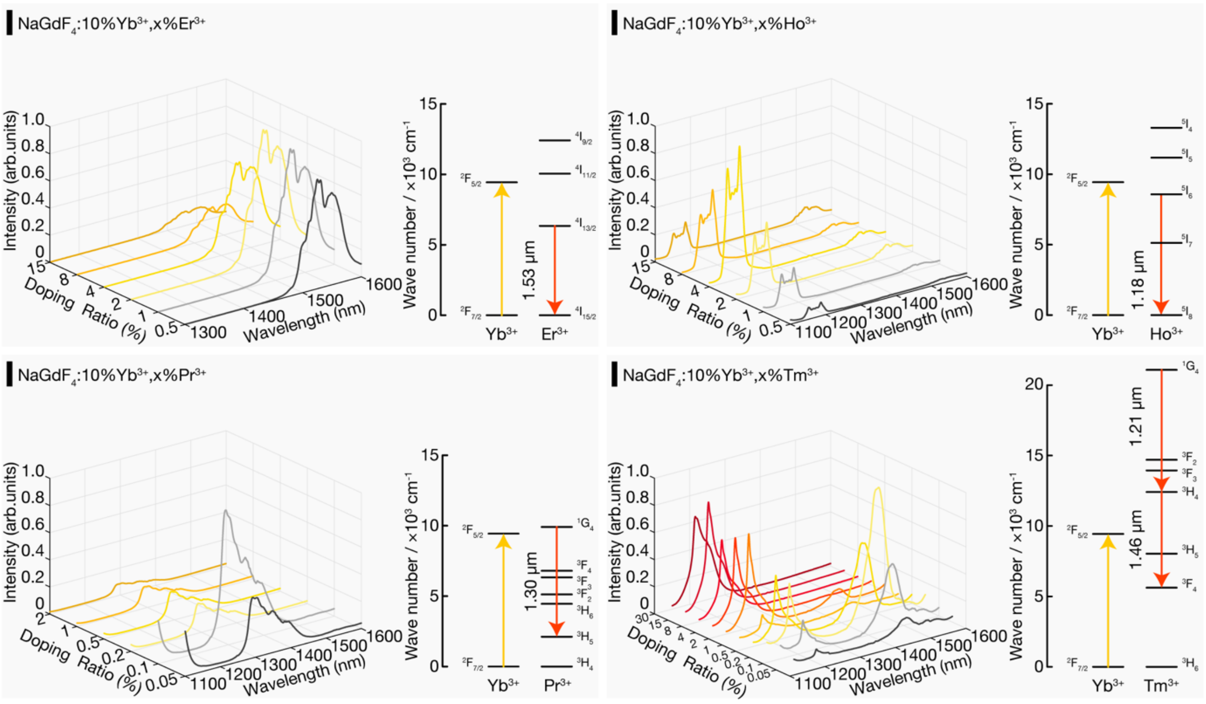

Supplementary Fig. 6 | Emission spectra (ex=980 nm) of $NaGdF_4$:10%Yb^{3+},*x*%Ln^{3+} (Ln=Er, Ho, Pr, Tm) and energy-level diagrams between Yb^{3+} and Ln^{3+} (Ln=Er, Ho, Pr, Tm). These results showed that Yb^{3+} could sensitize the emissions of Er^{3+}, Ho^{3+}, Pr^{3+}, and Tm^{3+}.

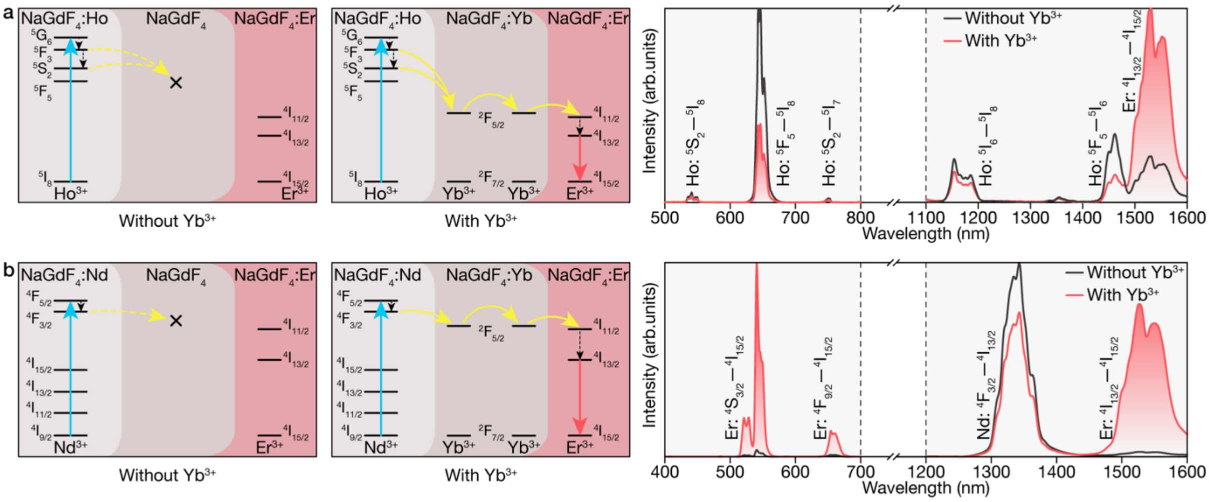

Supplementary Fig. 7 | a,b, Emissions of two model structures without or with Yb^{3+}-mediated ET (**a**) from Ho^{3+} to Er^{3+} (ex=448 nm) and (**b**) from Nd^{3+} to Er^{3+} (ex=808 nm) in core/shell/shell NCs.

Remarkable enhancement of Er^{3+} emissions at both visible (540 nm) and NIR region (1527 nm) was detected for Nd^{3+}-Yb^{3+}-Er^{3+} case (**b**). While the enhancement of Er^{3+} emissions was only observed at the NIR region for the Ho^{3+}-Yb^{3+}-Er^{3+} case. Since both Ho^{3+} and Er^{3+} had fluorescence at ~540 nm, for $NaGdF_4$:Ho@$NaGdF_4$:Yb@$NaGdF_4$:Er NCs, the visible light emission might mainly come from Ho under the excitation of 448 nm (absorption of Ho). So, the fluorescence intensity of Ho would decrease when Ho has an extra energy transfer to Yb.

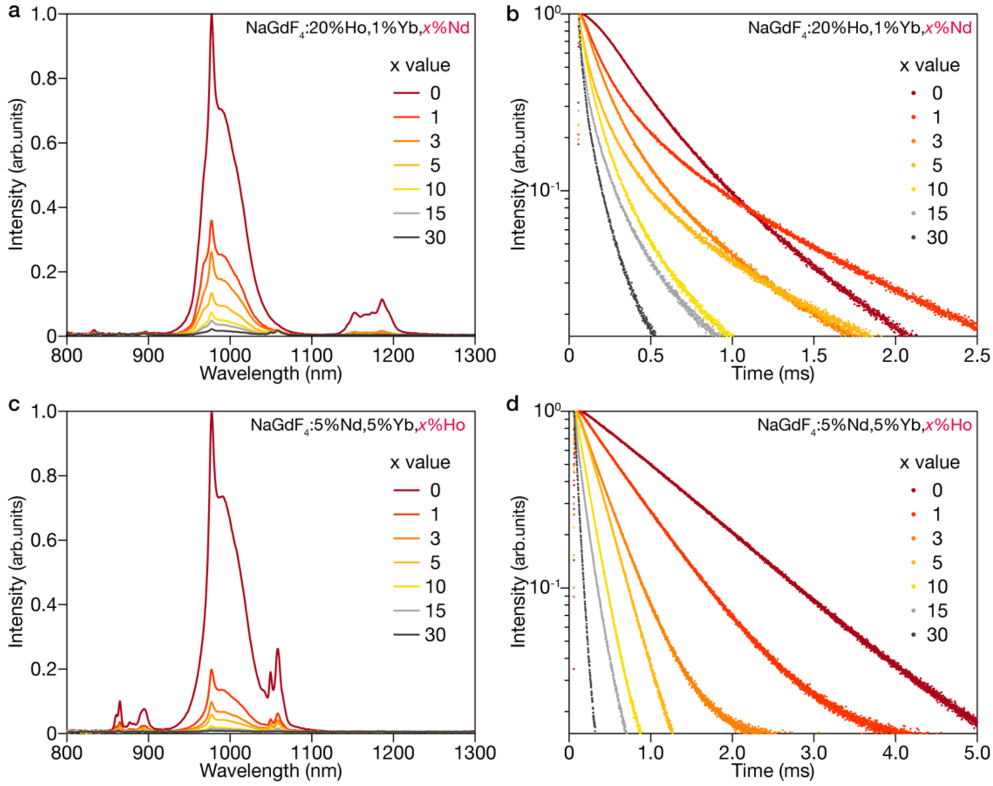

Supplementary Fig. 8 | a, Emission spectra of $NaGdF_4$:20%Ho^{3+},1%Yb^{3+},*x*%Nd^{3+} (x=0, 1, 3, 5, 10, 15 and 30) (ex: 448 nm). **b**, Fluorescence decay curves (monitoring the emission of Yb^{3+} at 978 nm) of the samples in **a**. **c**, Emission spectra of $NaGdF_4$:5%Nd^{3+},5%Yb^{3+},*x*%Ho^{3+} (x=0, 1, 3, 5, 10, 15 and 30) (ex: 574 nm). **d**, Fluorescence decay curves (monitoring the emission of Yb^{3+} at 978 nm) of the samples in **c.**

Supplementary Fig. 8a showed that the introduction of Nd^{3+} in $NaGdF_4$:20%Ho,1%Yb caused a strong quenching effect for the energy transfer from Ho^{3+} to Yb^{3+}. And the introduction of Ho^{3+} in $NaGdF_4$:5%Nd,5%Yb also imparted a similar effect (Supplementary Fig. 8c). This phenomenon was attributed to the cross-relaxation between Ho^{3+} and Nd^{3+}, which resulted in extra energy loss of sensitizers and thus the decrease of the lifetime of the excited state of Yb^{3+} (Supplementary Fig. 8b,d).

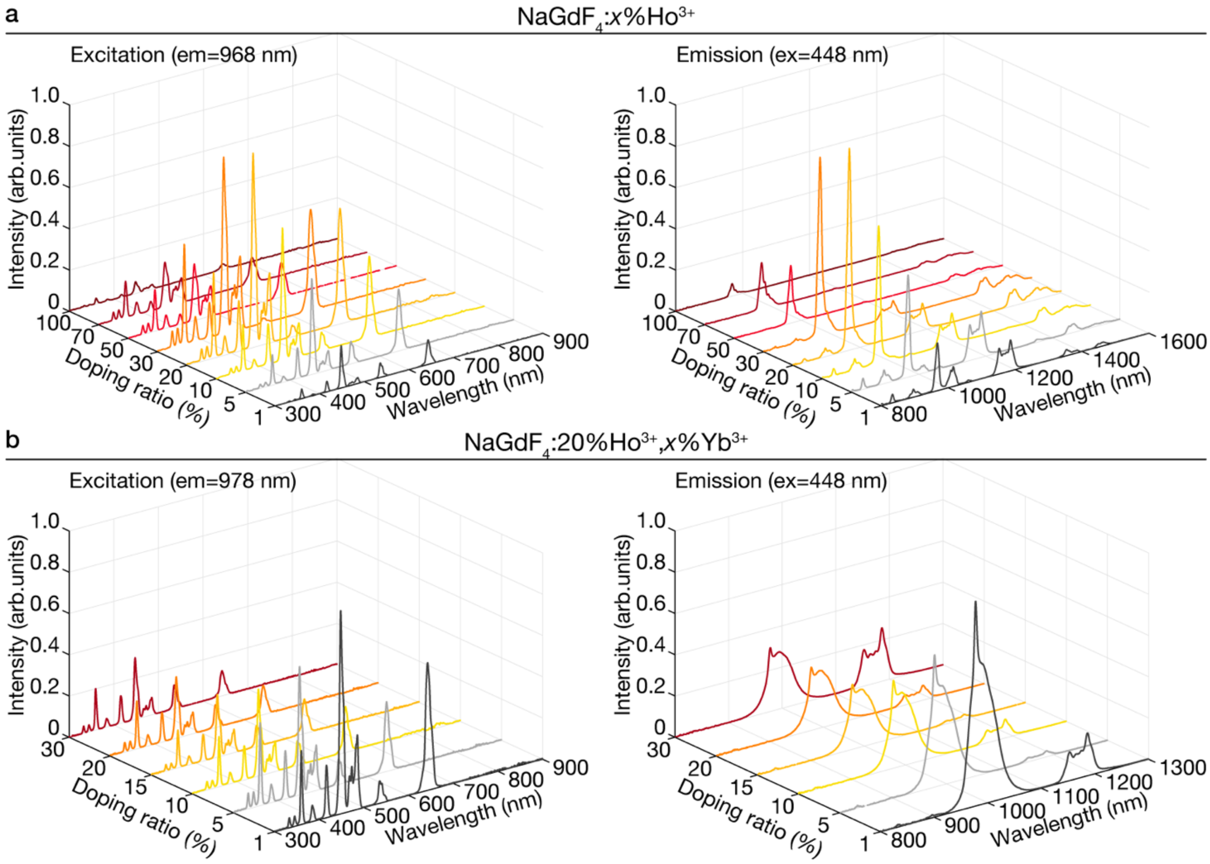

Supplementary Fig. 9 | a, Excitation and emission spectra of $NaGdF_4$:*x*%Ho^{3+}. **b**, Excitation and emission spectra of $NaGdF_4$:20%Ho^{3+},*x*%Yb^{3+}.

Supplementary Fig. 9a showed that the optimal doping ratio of Ho^{3+} for $NaGdF_4$:*x*%Ho^{3+} was 20%, and Supplementary Fig. 9b showed that the optimal doping ratio of Yb^{3+} for $NaGdF_4$:20%Ho^{3+},*x*%Yb^{3+} was 1%.

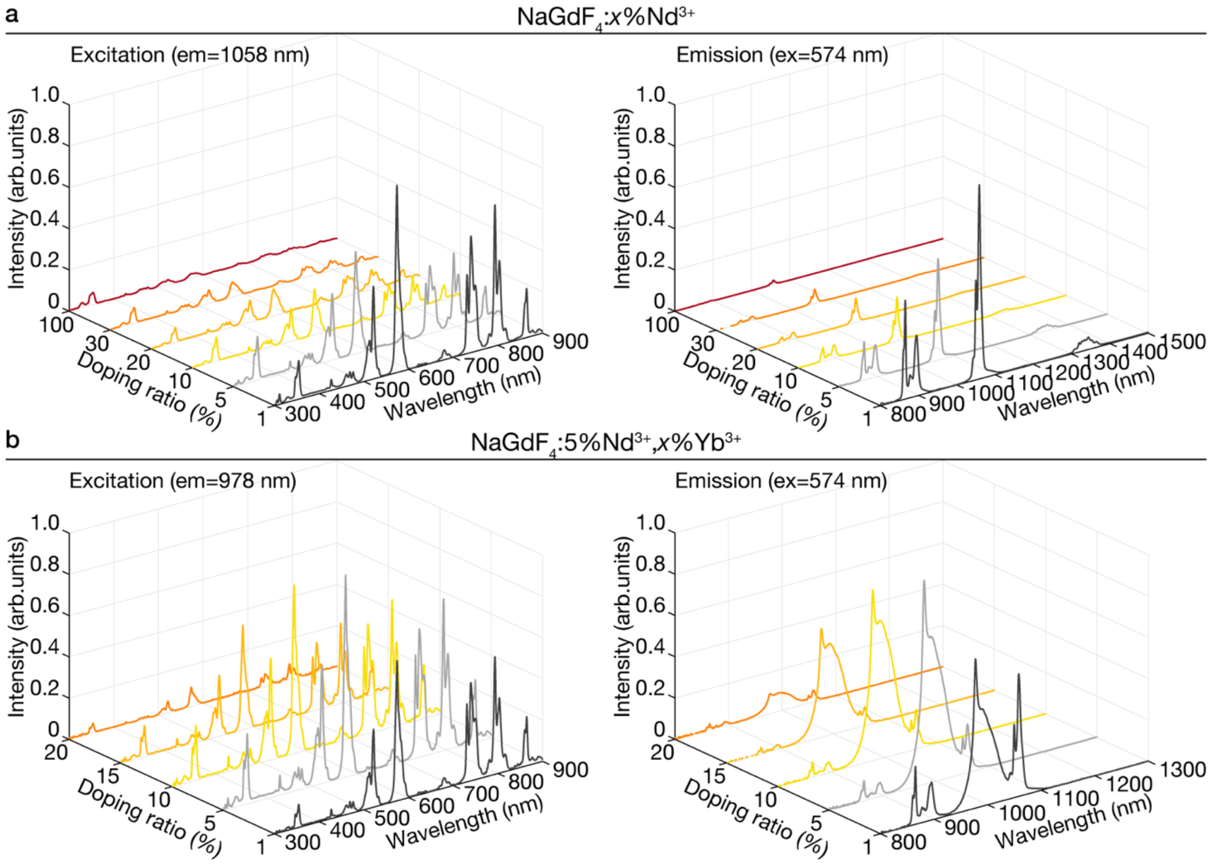

Supplementary Fig. 10 | a, Excitation and emission spectra of $NaGdF_4$:*x*%Nd^{3+}. **b**, Excitation and emission spectra of $NaGdF_4$:5%Nd^{3+},*x*%Yb^{3+}.

Supplementary Fig. 10a showed that the optimal doping ratio of Nd^{3+} for $NaGdF_4$:*x*%Nd^{3+} was 1%. But we finally chose the ratio of 5%, as this ratio gave rise to a more uniform distribution of excitation intensities. Then the optimal doping ratio of Yb^{3+} for $NaGdF_4$:5%Nd^{3+},*x*%Yb^{3+} was determined to be 5%, as shown in Supplementary Fig. 10b.

Supplementary Table 1 | Precursors, amounts and solvents for the preparation of penta-layer core-shell NCs.

NCs	Chemical formula	Precursors	Amounts (mmol)	Solvents
C	$NaHoF_4$	Na(TFA)	2.00	OA (10 mL)
		$Ho(TFA)_3$	1.00	ODE (10 mL)
CS	$NaGdF_4$:20%Ho,1%Yb	Na(TFA)	1.80	OA (4 mL)
		$Gd(TFA)_3$	0.79	ODE (6 mL)
		$Ho(TFA)_3$	0.20	
		$Yb(TFA)_3$	0.01	
CSS	$NaGdF_4$:10%Yb,2%Er<br>($NaGdF_4$:10%Yb,4%Ho)<br>($NaGdF_4$:10%Yb,0.1%Pr)<br>($NaGdF_4$:10%Yb,0.2%Tm)	Na(TFA)	2.70	OA (6 mL)
		$Gd(TFA)_3$	1.32	ODE (9 mL)
		$Yb(TFA)_3$	0.15	
		$Er(TFA)_3$	0.03	
CSSS	$NaGdF_4$:5%Nd,5%Yb	Na(TFA)	2.70	OA (6 mL)
		$Gd(TFA)_3$	1.35	ODE (9 mL)
		$Nd(TFA)_3$	0.075	
		$Yb(TFA)_3$	0.075	
CSSSS	$NaGdF_4$	Na(TFA)	0.90	OA (2 mL)
		$Gd(TFA)_3$	0.50	ODE (3 mL)

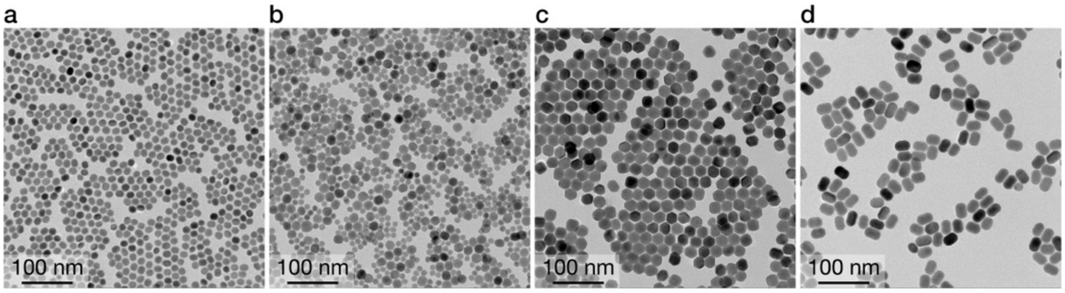

Supplementary Fig. 11 | a, TEM image of core NCs ($NaHoF_4$). **b-d**, TEM images of $NaHoF_4$@$NaGdF_4$ NCs using different coating schemes (*T*, temperature; *v*, feed rate of $NaGdF_4$ precursor): **b**, *T*=280 °C, *v*=1.0 mmol h^{-1}. **c**, *T*=280 °C, *v*=0.6 mmol h^{-1}. **d**, *T*=290 °C, *v*=0.6 mmol h^{-1}.

Supplementary Fig. 11b showed that the quick injection of precursors could result in the autonucleation of precursors, as evidenced by the appearance of smaller NCs. And Supplementary Fig. 11c,d showed that higher temperature might cause the evolution of morphology from equiaxed to rod-shaped.

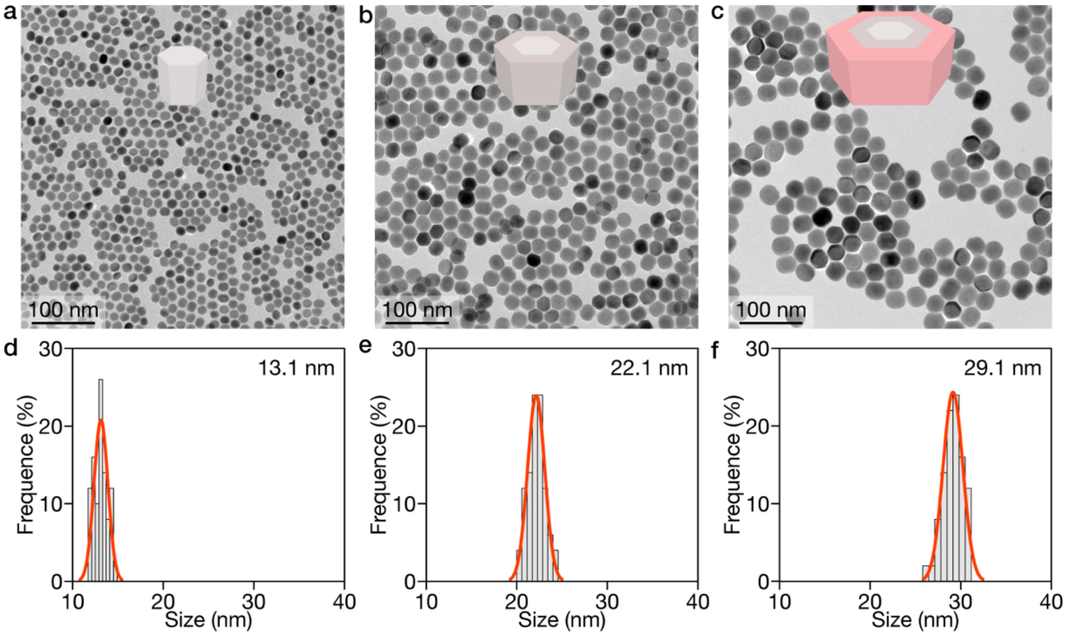

Supplementary Fig. 12 | a-c, TEM images of **a**, core NCs; **b**, CS NCs; **c**, CSS NCs. **d-f**, Size distributions of **d**, core NCs; **e**, CS NCs; **f**, CSS NCs. (The insets showed the cross sections of multi-layer core-shell nanostructure.)

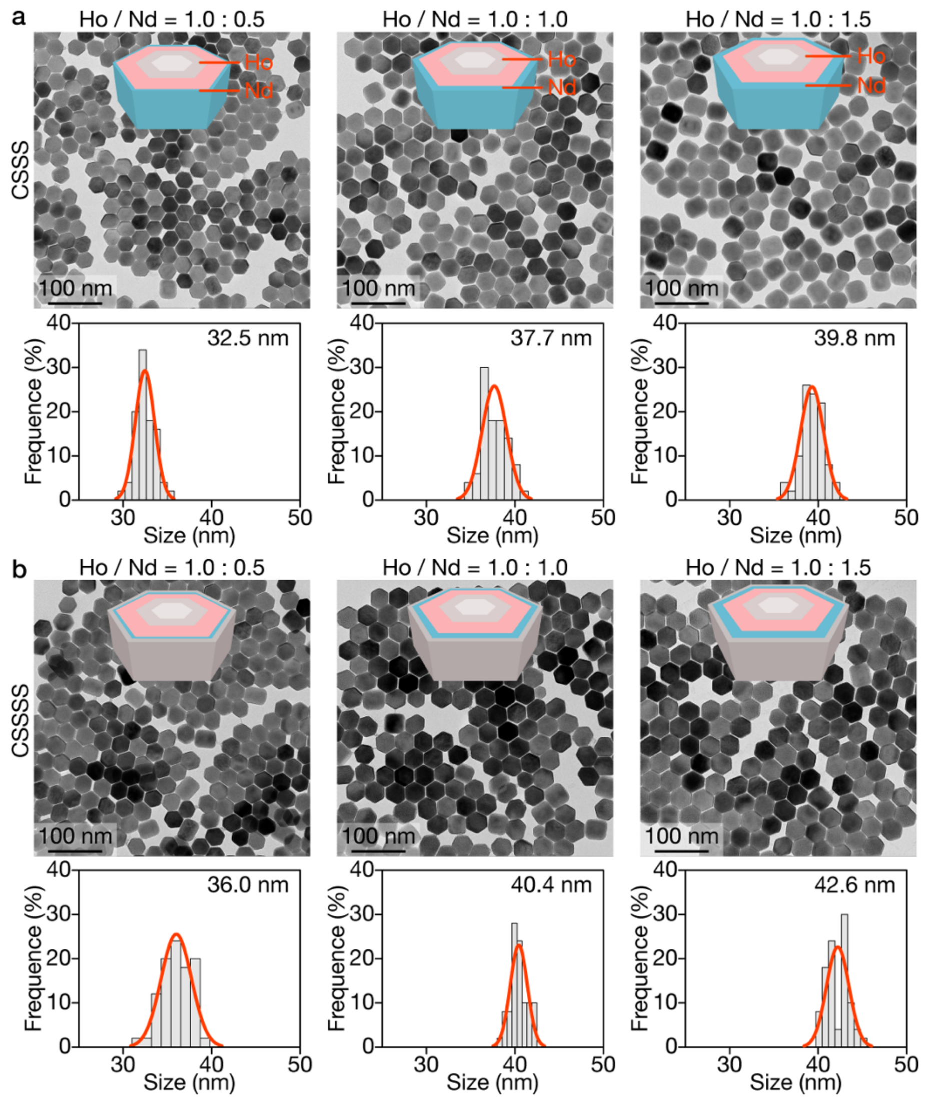

Supplementary Fig. 13 | a, TEM images and the corresponding size distributions of CSSS NCs with different Ho-layer/Nd-layer precursor ratios. **b**, TEM images and the corresponding size distributions of CSSSS NCs with different Ho-layer/Nd-layer precursor ratios. (The insets showed the cross sections of multi-layer core-shell nanostructure.)

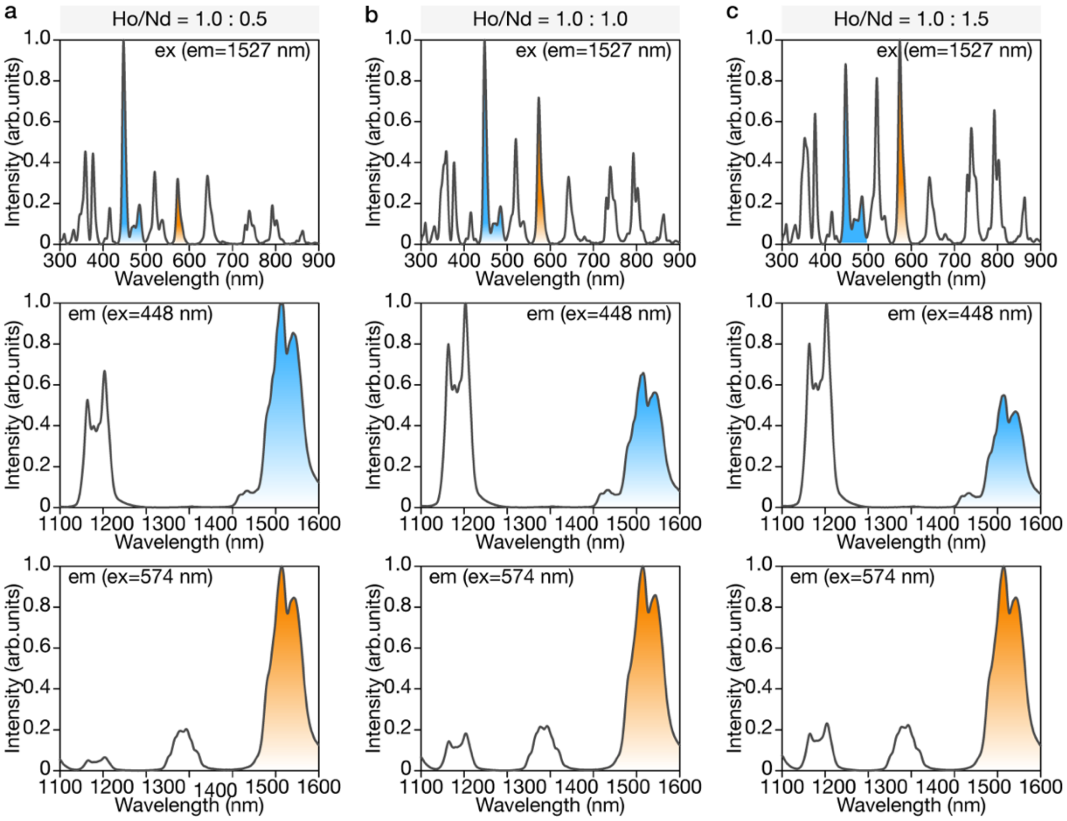

Supplementary Fig. 14 | Normalized excitation and emission spectra of Er-NCs (CSSSS) with different Ho-layer/Nd-layer precursor ratios. a, Ho/Nd=1.0:0.5. **b**, Ho/Nd=1.0:1.0. **c**, Ho/Nd=1.0:1.5.

The peaks marked with blue in excitation spectra represented the contribution of Ho, while that marked with orange represented the contribution of Nd. As the Ho-layer/Nd-layer precursor ratio varied from 1.0:0.5 to 1.0:1.5, the contribution of Nd increased gradually.

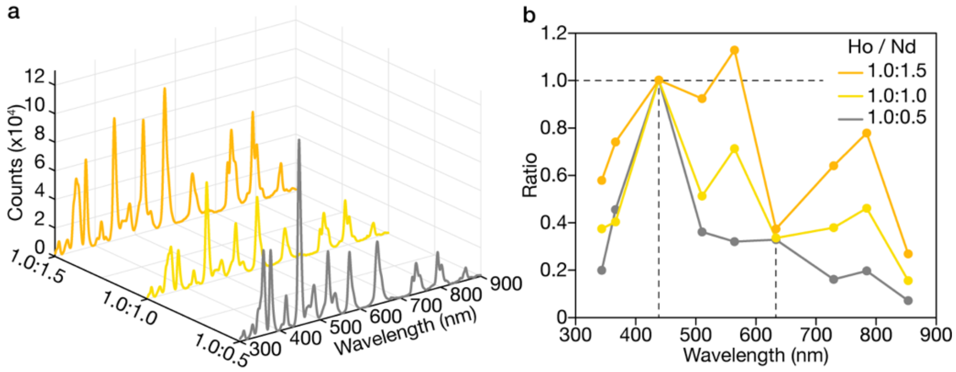

Supplementary Fig. 15 | a, Excitation (em=1527 nm) spectra of Er-NCs (CSSSS) with different Ho-layer/Nd-layer precursor ratio. **b**, Normalized excitation intensities at 353, 377, 448, 520, 574, 643, 739, 793, and 863 nm for different Ho-layer/Nd-layer precursor ratios (normalized to their respective intensity at 448 nm).

The intensities at 448 nm and 643 nm were contributed by Ho. During this coating process, the relative contribution of Nd increased almost proportionally to its precursor amount, while that of Ho remained nearly unchanged.

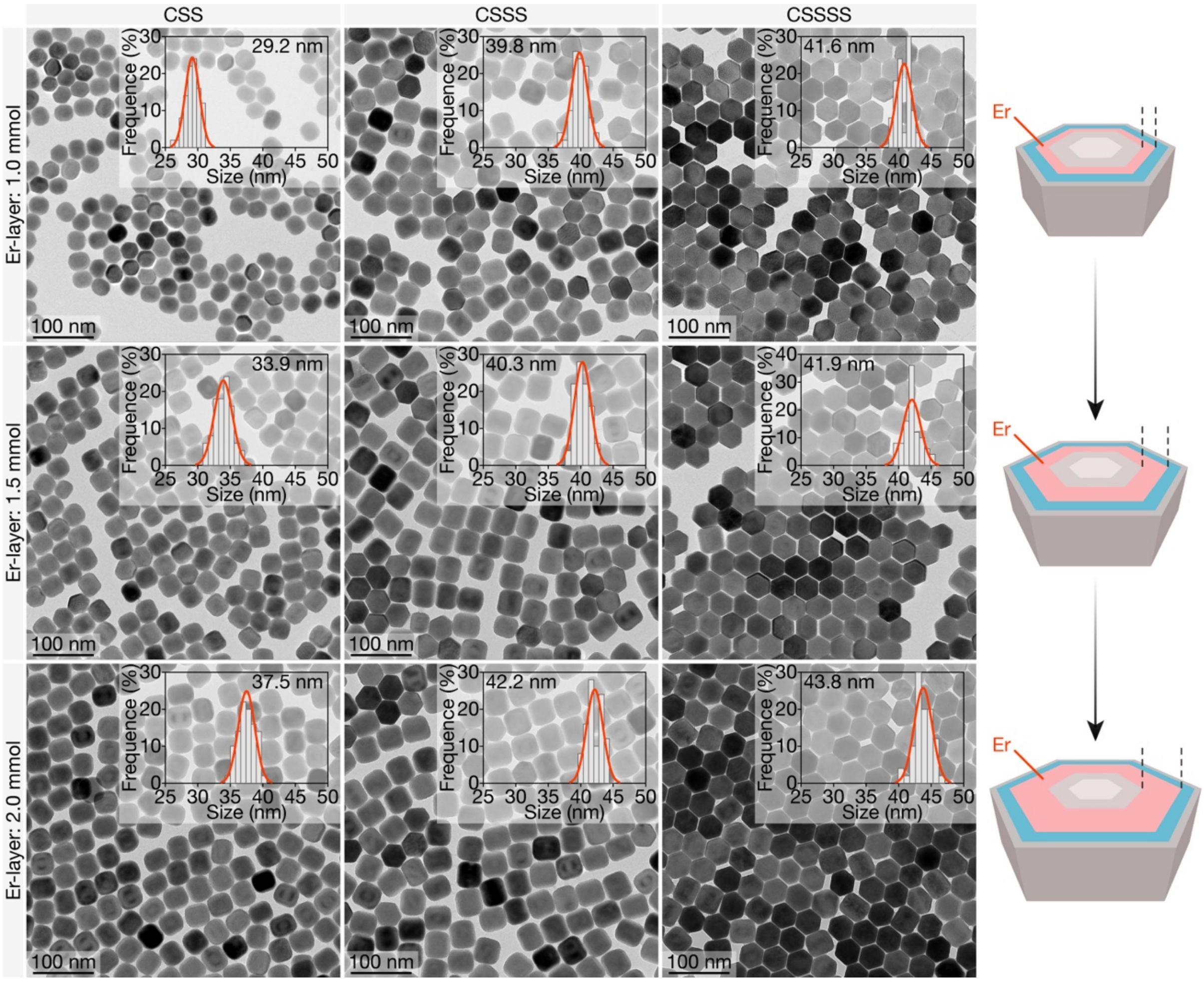

Supplementary Fig. 16 | TEM images and their size distributions of CSS, CSSS, and CSSSS NCs with different precursor amounts of Er-layer. (The insets showed the cross sections of multi-layer core-shell nanostructure of CSSSS NCs.)

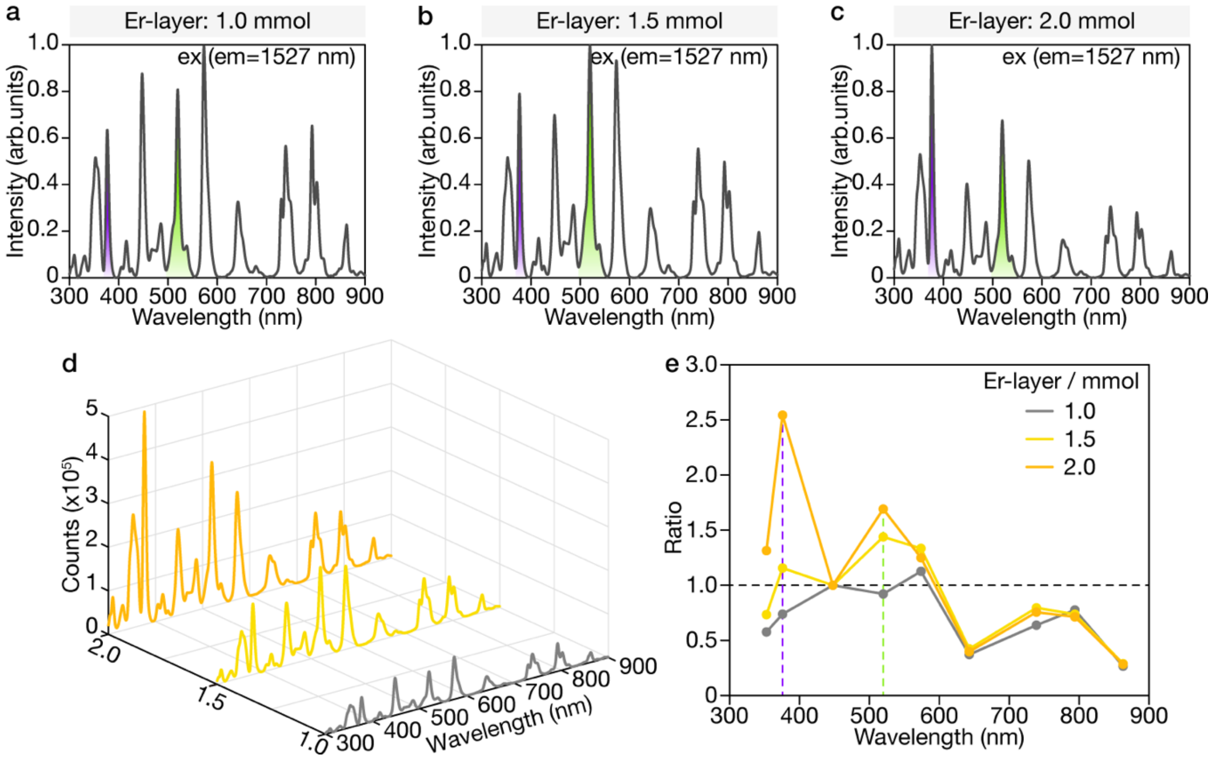

Supplementary Fig. 17 | Normalized excitation (em=1527 nm) spectra of Er-NCs (CSSSS) with different precursor amounts of Er-layer: **a**, 1.0 mmol; **b**, 1.5 mmol; **c**, 2.0 mmol. **d**, Comparison of three excitation spectra. **e**, Normalized excitation intensities at 353, 377, 448, 520, 574, 643, 739, 793, and 863 nm for different precursor amounts of Er-layer (normalized to their respective intensity at 448 nm).

With the increase of precursor amount of Er-layer, the emission increased. But the relative intensity of each peak kept nearly unchanged. This phenomenon showed a characteristic of the nanostructure, i.e., the independence of excitation with emission. Note that the intensities at 377 nm and 520 nm (partially) were contributed by Er (marked with purple and green respectively in Supplementary Fig. 17a-c). So, during this coating process, only these two sites showed distinct increases in excitation intensity (Supplementary Fig. 17e). We finally chose a precursor amount of 1.5 mmol, since this guaranteed the overall shape of the excitation spectrum, meanwhile improved the intensity of NCs.

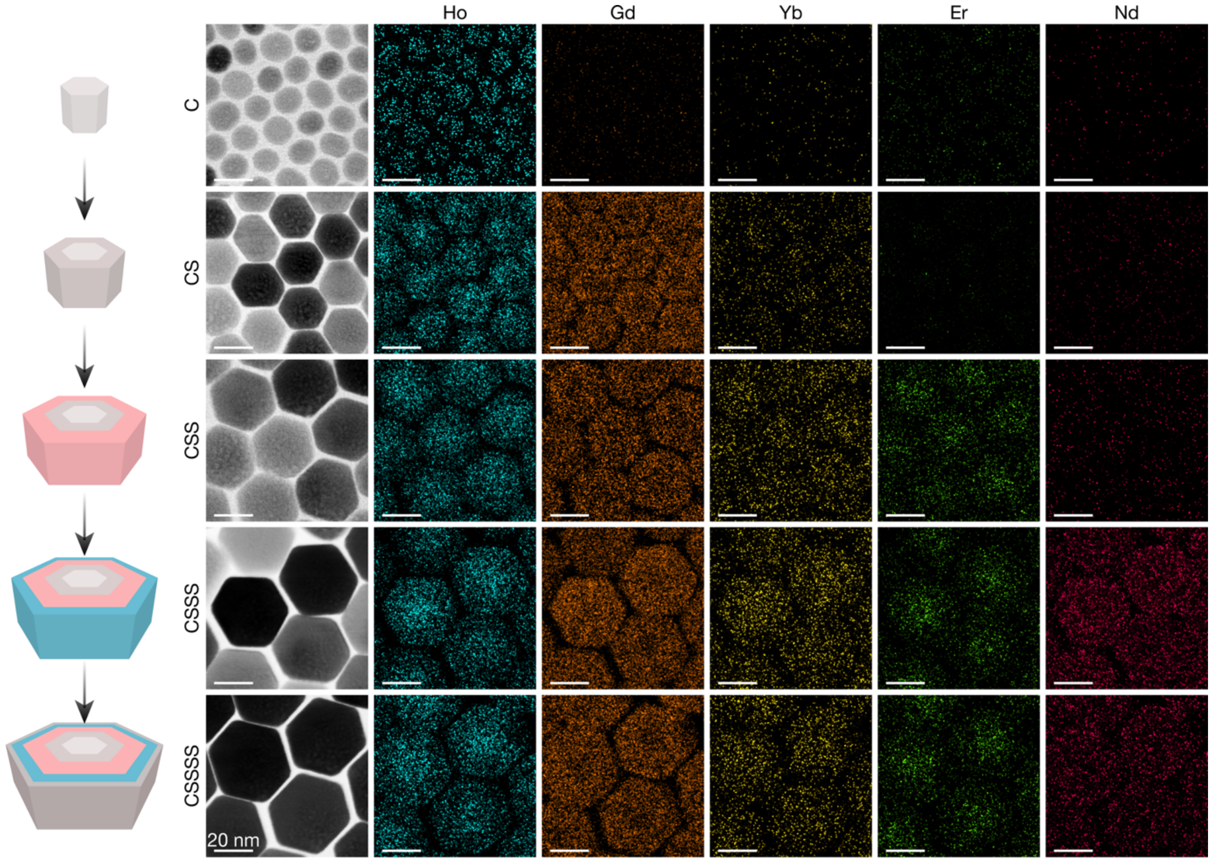

Supplementary Fig. 18 | STEM-EDS elemental mapping of C, CS, CSS, CSSS, and CSSSS NCs (Er^{3+} as the activator). (The illustration of NCs showed the cross sections of multi-layer core-shell nanostructure.)

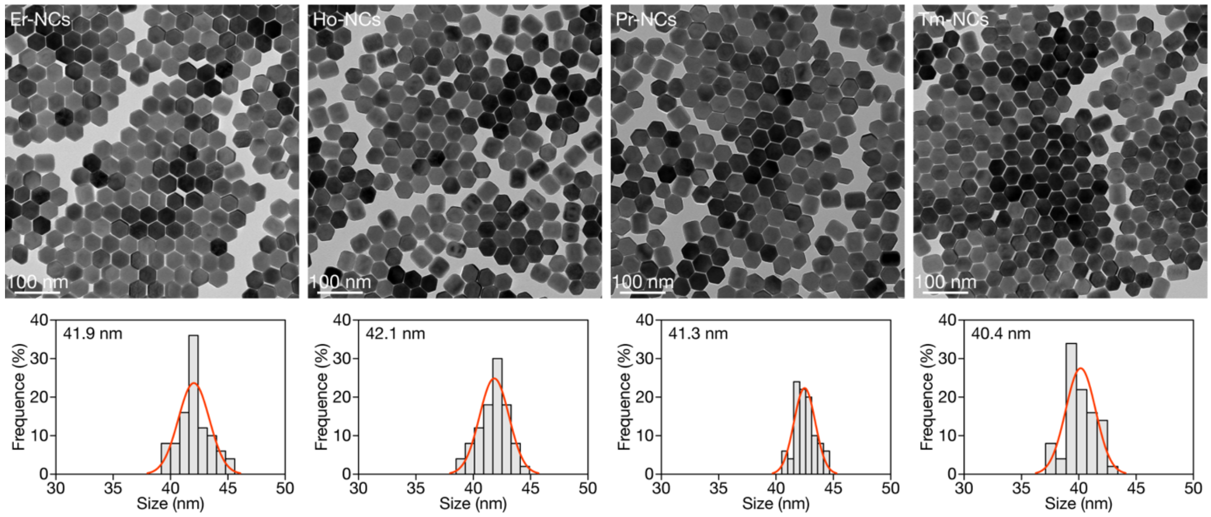

Supplementary Fig. 19 | TEM images and the corresponding size distributions of Er/Ho/Pr/Tm-NCs.

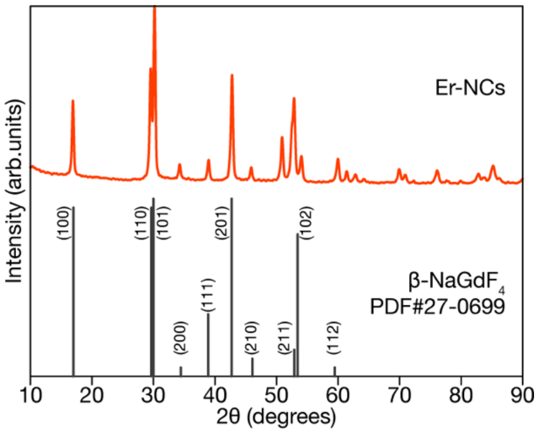

Supplementary Fig. 20 | XRD pattern of the as-prepared Er-NCs and the standard diffraction pattern of β-$NaGdF_4$.

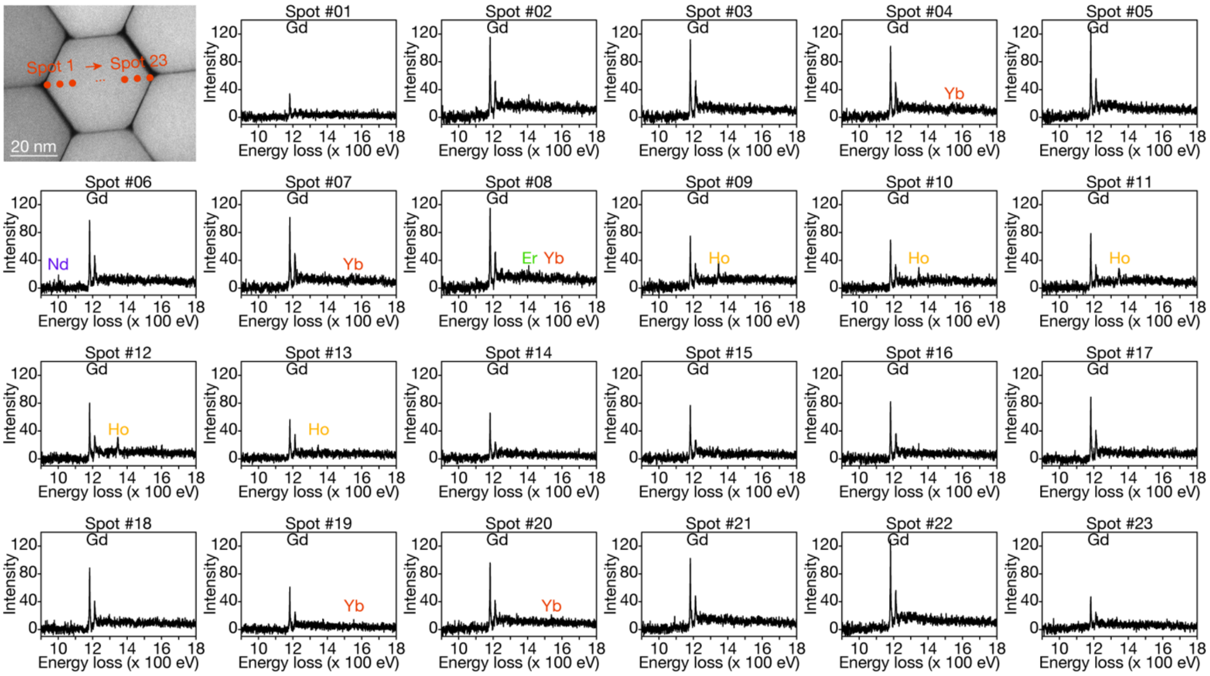

Supplementary Fig. 21 | Electron energy loss spectroscopy spectra collected from spots 1 to 23 on the cross-section of a single Er-NC.

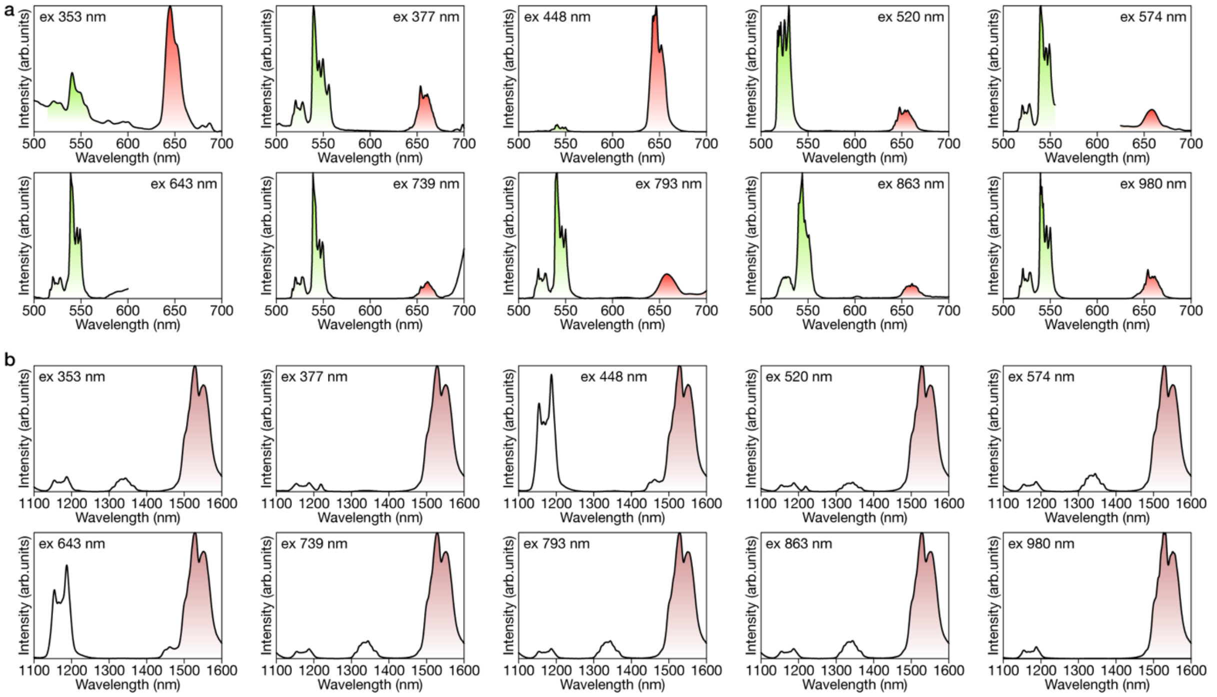

Supplementary Fig. 22 | a, Visible emission spectra of Er-NCs under various excitations. **b**, NIR emission spectra of Er-NCs under various excitations.

The relatively stronger red emission (646 nm) of Er-NCs under the excitation corresponding to the absorption of Ho^{3+} (353 and 448 nm) might be attributed to the infiltration of Ho^{3+} emission, as Ho^{3+} had nearly overlapping emission at 540 nm ($^5S_2$-$^5I_8$) and 646 nm ($^5F_5$-$^5I_8$) with that of Er^{3+}.

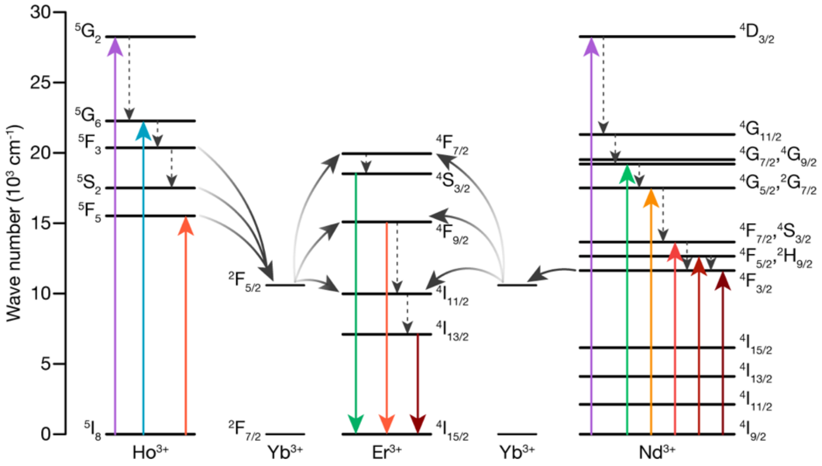

Supplementary Fig. 23 | Transitions of energy levels corresponding to major absorptions and the visible/NIR emissions of Er-NCs.

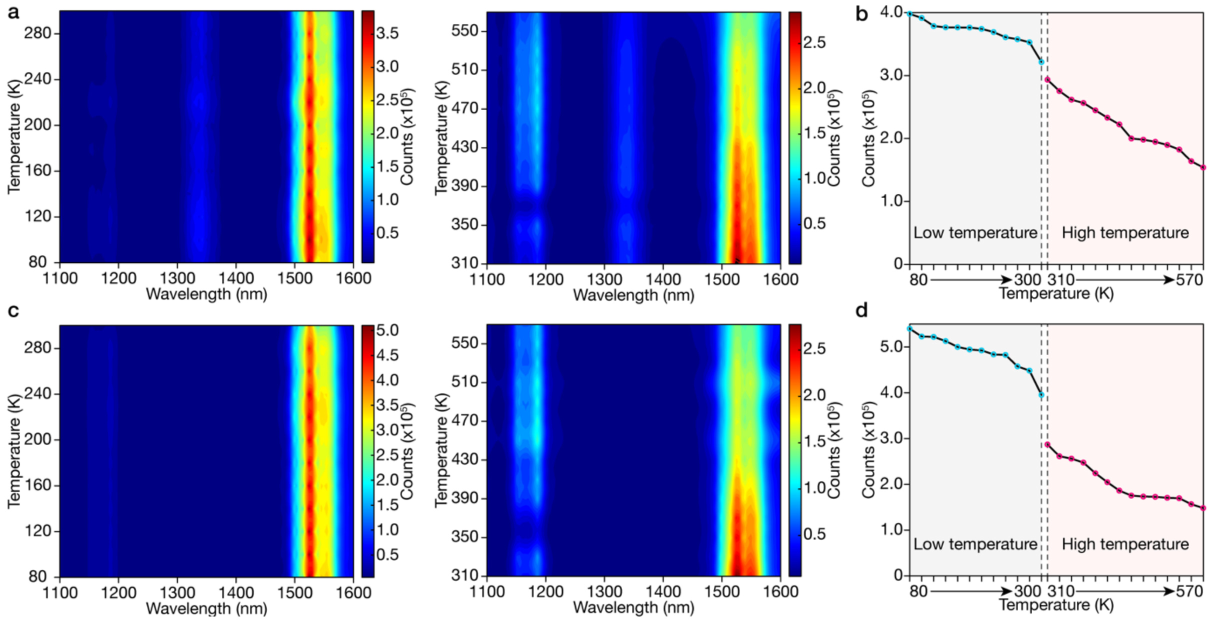

Supplementary Fig. 24 | a, Temperature-dependent photoluminescence maps under the excitation of the 808-nm laser. **b**, Normalized intensity of Er emission (1527 nm) in **a**. **c**, Temperature-dependent photoluminescence maps under the excitation of a 980-nm laser. **d**, Normalized intensity of Er emission (1527 nm) in **c**. (Note that the measurements of the high-temperature part and low-temperature part were performed in different optical paths.)

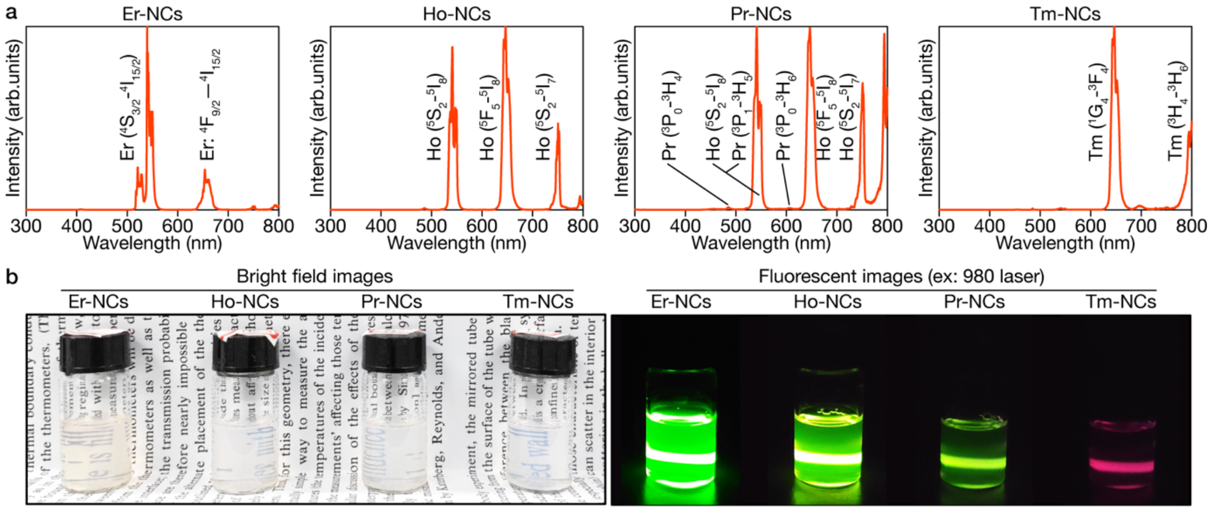

Supplementary Fig. 25 | a, Visible emission spectra of the as-prepared Er/Ho/Pr/Tm-NCs (ex: 980-nm laser). **b**, Bright-field images (left) and fluorescent images of the as-prepared Er/Ho/Pr/Tm-NCs under the irradiation of a 980-nm laser (right).

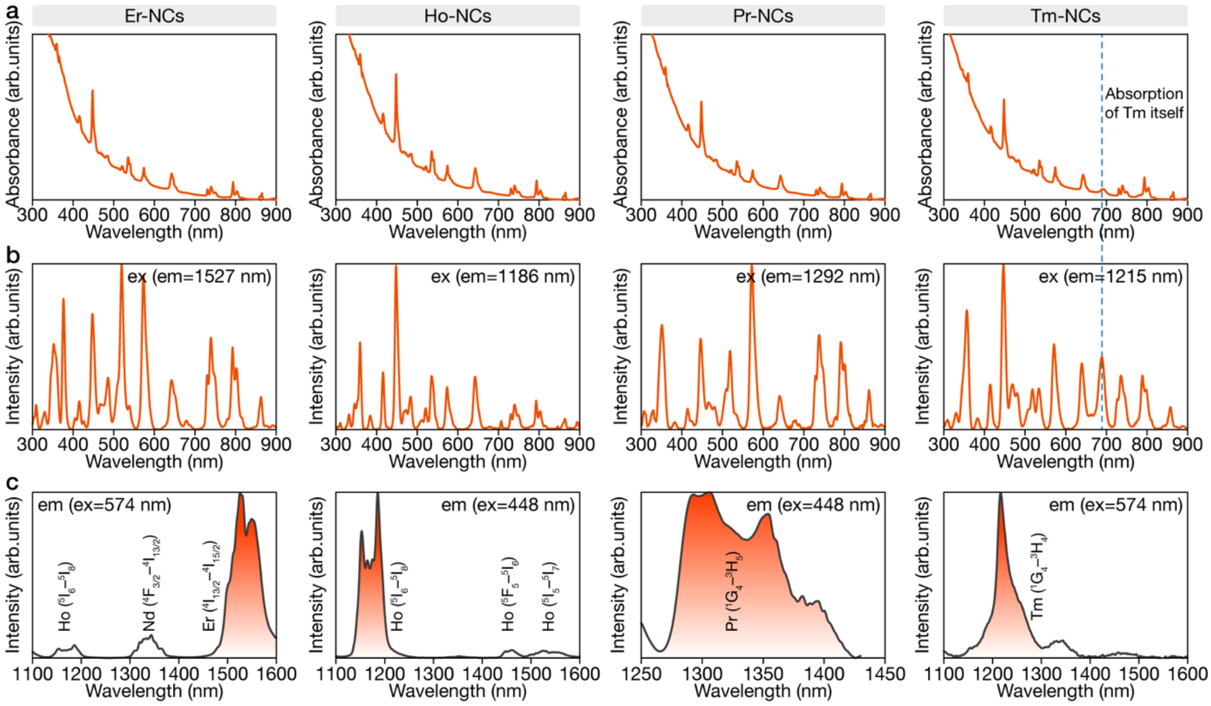

Supplementary Fig. 26 | a, Absorption, **b**, excitation and **c**, NIR-emission spectra of Er/Ho/Pr/Tm-NCs.

The relatively higher absorbance of Ho-NCs at 448 nm and 643 nm was due to the double contribution of Ho from the sensitizer layer and activator layer. The appearance of the peak at 690 nm on the absorption and excitation spectra of Tm-NCs was ascribed to the absorption of Tm itself (Supplementary Fig. 2). Also, note that the excitation spectra of Pr- and Tm-NCs might not be all contributed by their own luminescence, as the emissions of Ho^{3+} (1186 nm) and Nd^{3+} (1330 nm) could overlap with that of Tm^{3+} (1215 nm) and Pr^{3+} (1292 nm), respectively (absorption layers contained Ho^{3+} and Nd^{3+}).

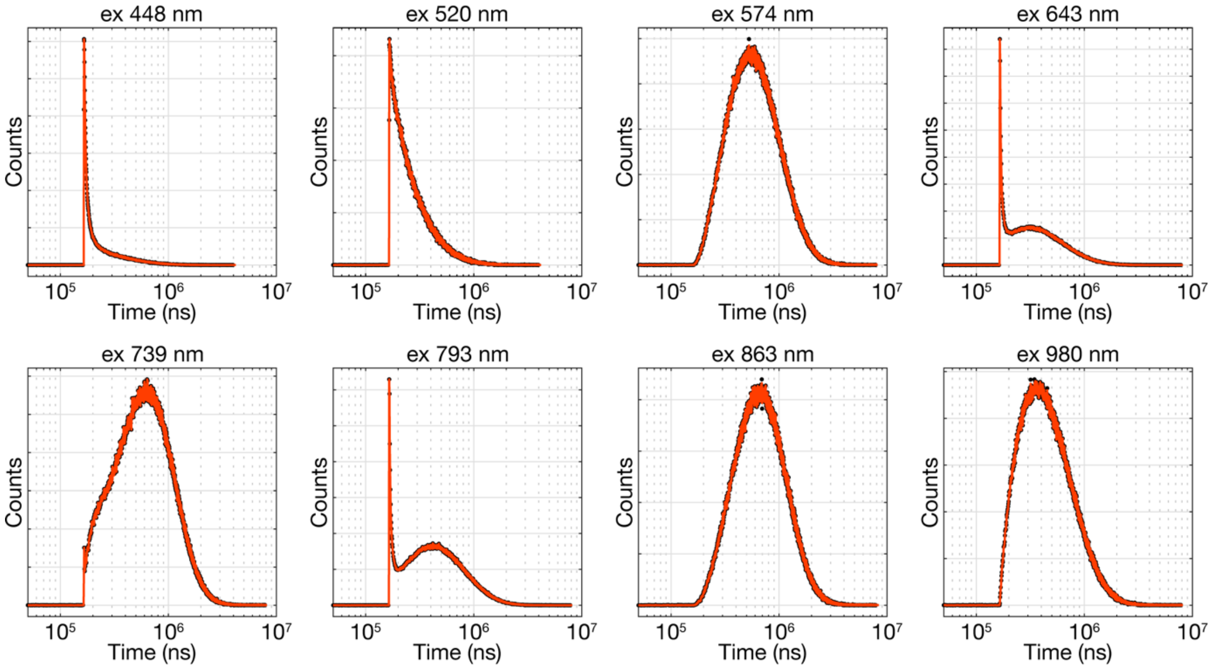

Supplementary Fig. 27 | Transient spectra of Er-NCs by monitoring the emission at 540 nm (the transient spectra excited by 353, 359, and 377 nm were not provided, as the relative weak energy of the OPO laser in this wavelength range was insufficient to excite the green fluorescence of Er-NCs).

Under different wavelengths of excitations, the curves showed distinct profiles. 448 nm and 643 nm of excitations were absorbed by Ho^{3+} (Supplementary Fig. 2). Ho^{3+} itself had an entirely overlapped emission with Er^{3+} at 540 nm, and the decay of Ho^{3+} at 540 nm was very fast. So, with 643 nm of excitation, the emission of Ho^{3+} resulted in the first rise-decay trail in the transient curve. With energy transferring from Ho^{3+} to Er^{3+}, the second rise-decay trail appeared. But for 448 nm of excitation, the curve only showed the decay trail of Ho^{3+}, which might be attributed to the very weak green fluorescence of the sample under the excitation of 448 nm (Supplementary Fig. 22a).

Excitation of 793 nm could be absorbed by Er^{3+} and Nd^{3+} (Supplementary Fig. 2). So Er^{3+} absorbed the energy from 793 nm excitation and yield the emission of 540 nm, the first rise-decay trail occurred. When the energy of 793 nm that was absorbed by Nd^{3+} was transferred to Er^{3+}, then the second rise-decay trail appeared.

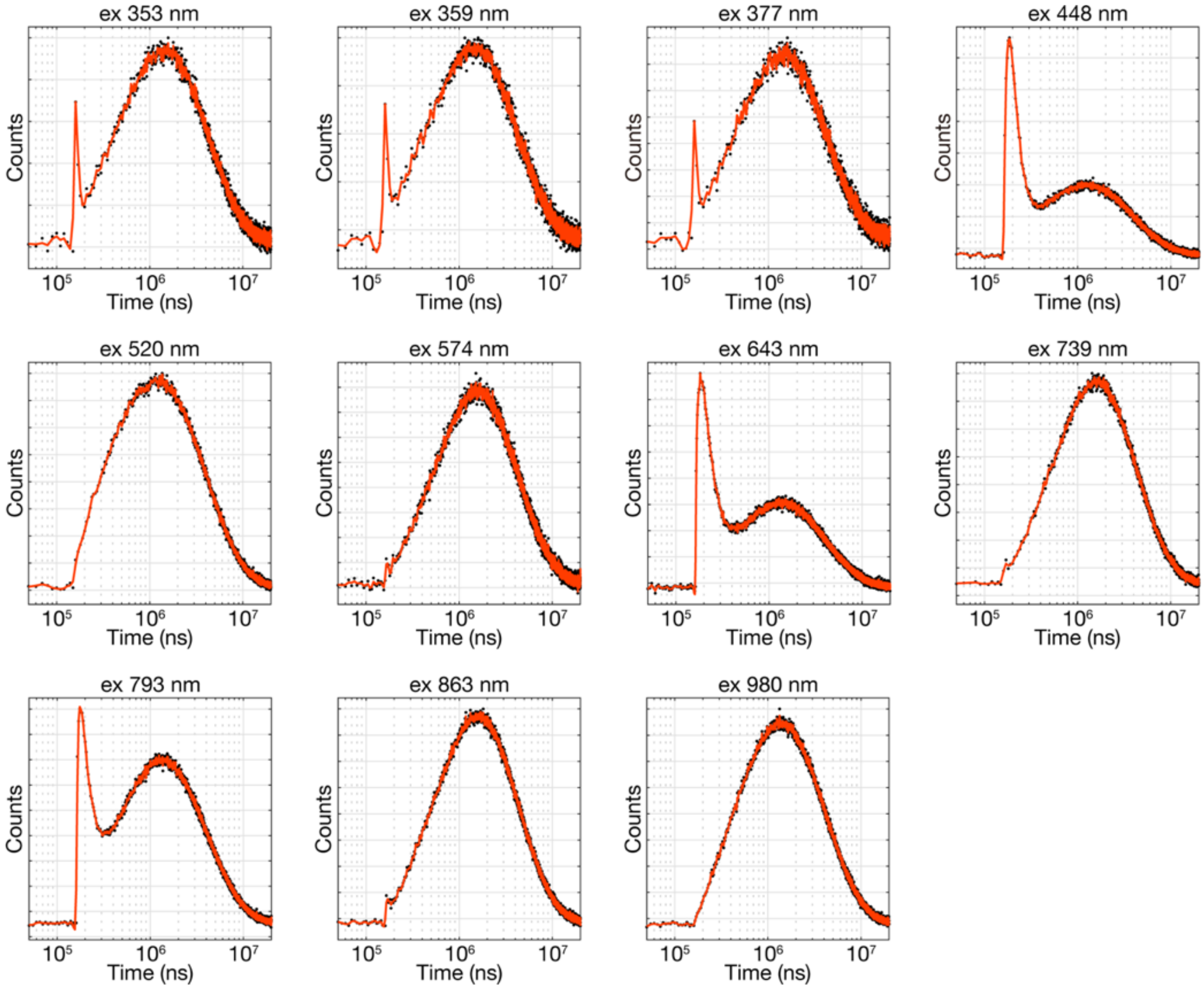

Supplementary Fig. 28 | Transient spectra of Er-NCs by monitoring the emission at 1527 nm.

The curves with excitations of 448 nm, 643 nm, and 793 nm showed a rise-decay-rise-decay trial. Among these, 448 nm and 643 nm of excitations were absorbed by Ho^{3+} (Supplementary Fig. 2). Ho^{3+} itself had an entirely overlapped emission with Er^{3+} at 1527 nm, and the decay of Ho^{3+} at 1527 nm was very fast. These two factors resulted in the first rise-decay trail. With energy transferring from Ho^{3+} to Er^{3+}, the second rise-decay trail appeared.

Excitation of 793 nm could be absorbed by Er^{3+} and Nd^{3+} (Supplementary Fig. 2). So Er^{3+} absorbed the energy of 793 nm and yield the emission of 1527 nm, the first rise-decay trail occurred. When the energy of 793 nm that was absorbed by Nd^{3+} was transferred to Er^{3+}, then the second rise-decay trail appeared.

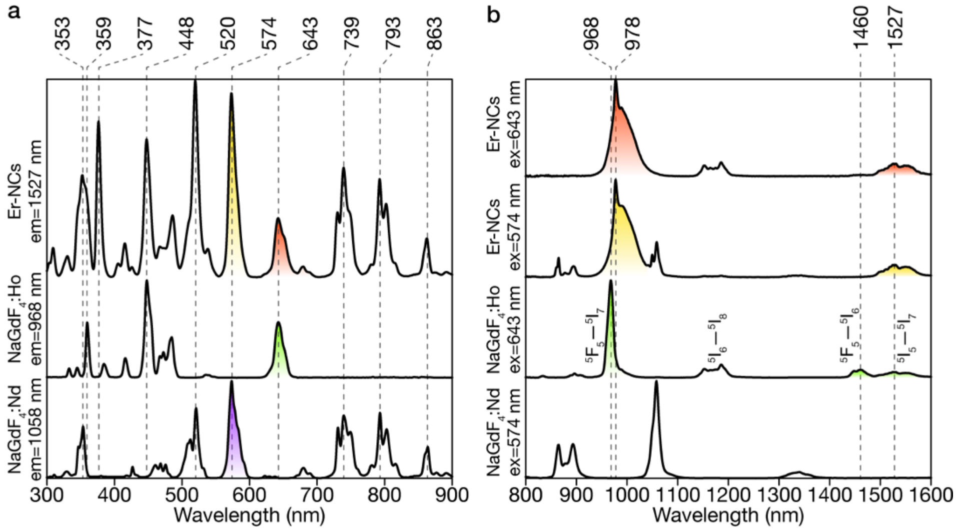

Supplementary Fig. 29 | a, Excitation spectra of the as-prepared Er-NCs, $NaGdF_4:Ho^{3+}$, and $NaGdF_4:Nd^{3+}$. **b**, Emission spectra of the as-prepared Er-NCs, $NaGdF_4:Ho^{3+}$, and $NaGdF_4:Nd^{3+}$.

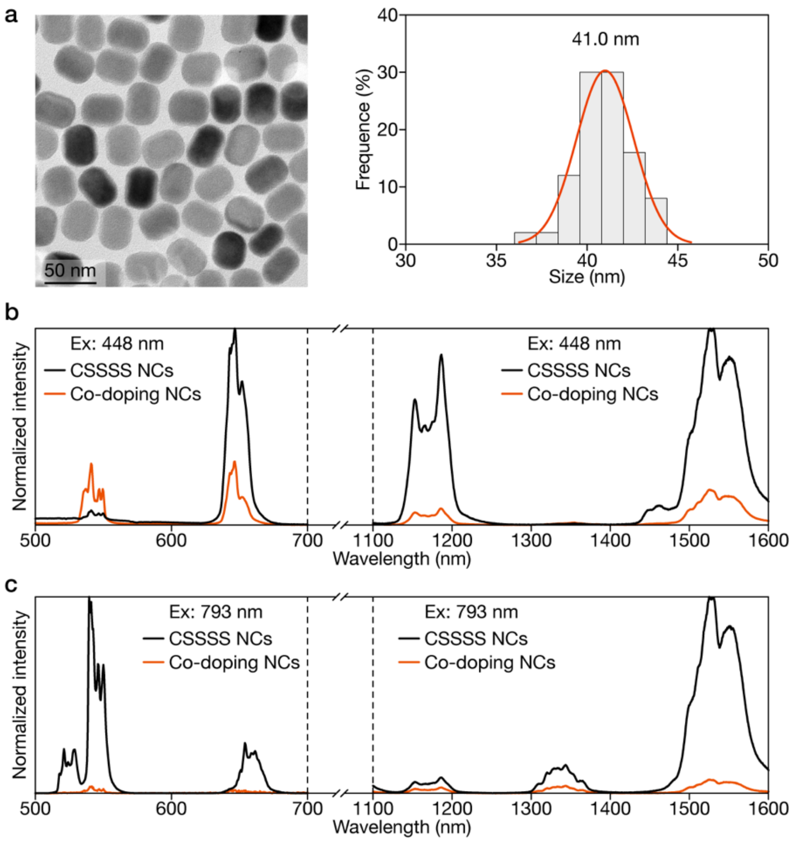

Supplementary Fig. 30 | a, TEM image and the size distribution of co-doping NCs. **b**, Emission spectra of co-doping NCs and CSSSS NCs under the excitation of 448 nm. **c**, Emission spectra of co-doping NCs and CSSSS NCs under the excitation of 793 nm.

The co-doping NCs showed notably reduced fluorescence intensity compared to that of CSSSS NCs. This deterioration was attributed to the cross-relaxation between Ho and Nd (Supplementary Fig. 8).

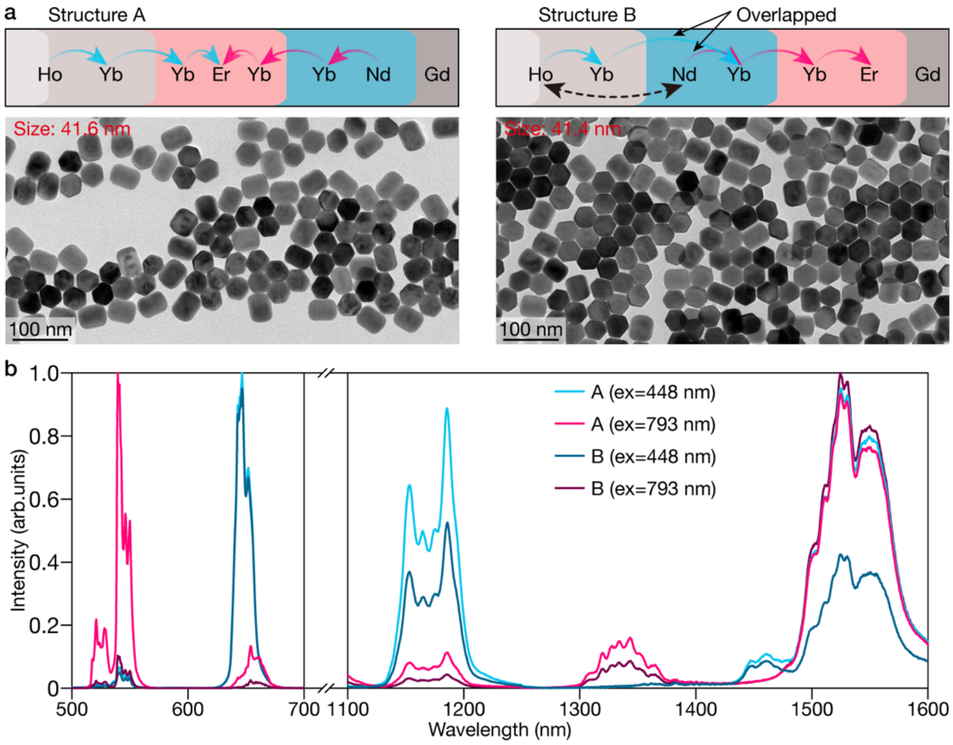

Supplementary Fig. 31 | a, Illustration of the nanostructures of Er-NCs with symmetric and asymmetric layouts of sensitizers, respectively, and their TEM images. **b**, Visible and NIR emission spectra of NCs (ex: 448 and 793 nm).

The symmetric structure of Er-NCs in Supplementary Fig. 31a was the same as that in Fig. 1c. For the asymmetric structure, the inner two layers were Ho-sensitizer-layers; the third layer was Nd-sensitizer-layer; the fourth layer was Er-activator-layer, and the fifth layer was $NaGdF_4$ inert shell. The samples were excited by two wavelengths of 448 and 793 nm, respectively, corresponding to the absorption of Ho and Nd.

In comparison to Er-NCs with symmetric structures, the asymmetric structure failed to avoid the overlap of energy flux, which caused severely decreased fluorescence in either the visible or NIR band. As shown in Supplementary Fig. 31b, under 448 nm-excitation, the visible emission kept almost unchanged, as the visible emission came mainly from Ho^{3+} itself; While the NIR emission halved since the energy transfer from Ho^{3+} to Er^{3+} was spatially impeded by the Nd-sensitizer-layer. Under 793 nm excitation, the NIR emission remained unchanged; While the visible emission decreased by 90%, which might be attributed to the quenching effect of Ho^{3+} to Nd^{3+}.

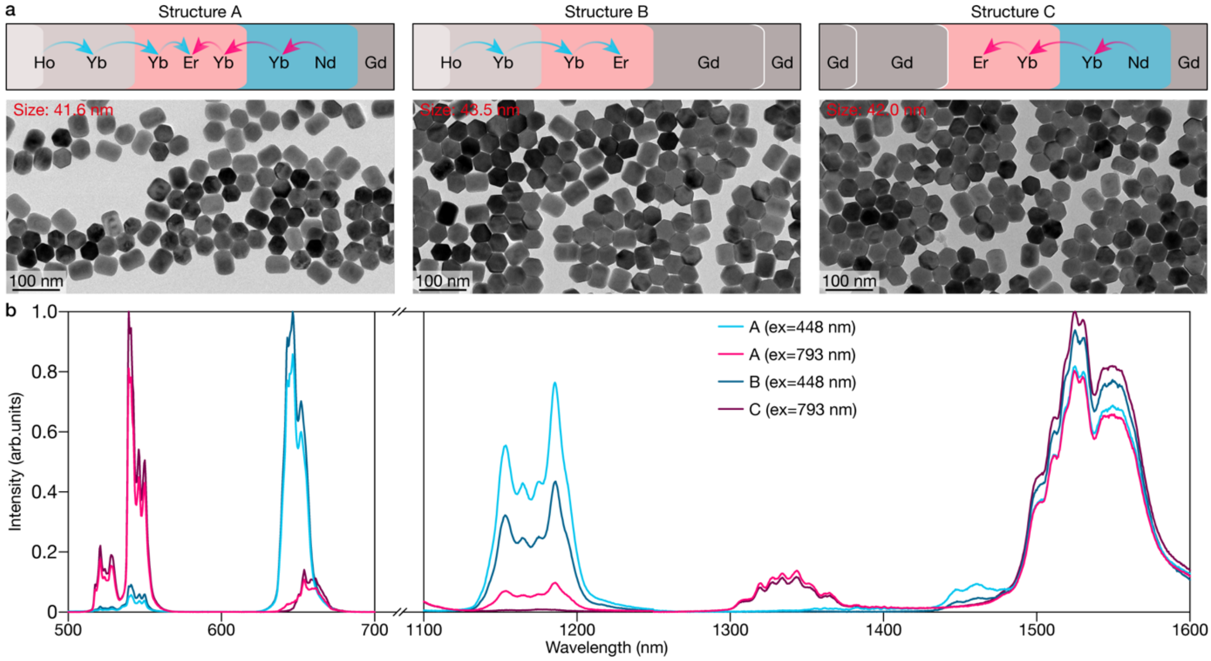

Supplementary Fig. 32 | a, Illustration of the nanostructure of Er-NCs with both Ho and Nd as sensitizers, and with either Ho or Nd as a sensitizer, respectively, and their TEM images. **b**, Visible and NIR emission spectra of NCs (ex: 448 and 793 nm).

The fluorescence of Er-NCs (Structure A) kept the same level with another two control samples with only either Ho-sensitizer (Structure B) or Nd-sensitizer (Structure C), meaning the symmetric structure successfully integrated Ho and Nd into a powerful sensitizer system while effectively eliminated their mutual deterioration.

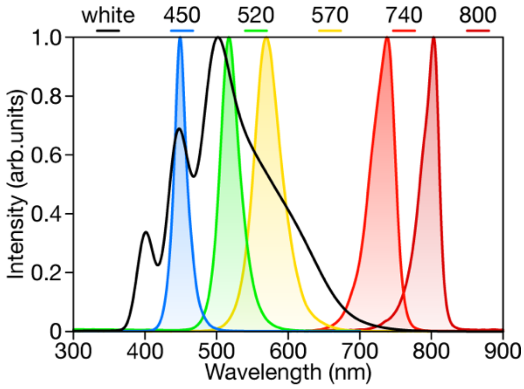

Supplementary Fig. 33 | Emission spectra of LEDs used in this study.

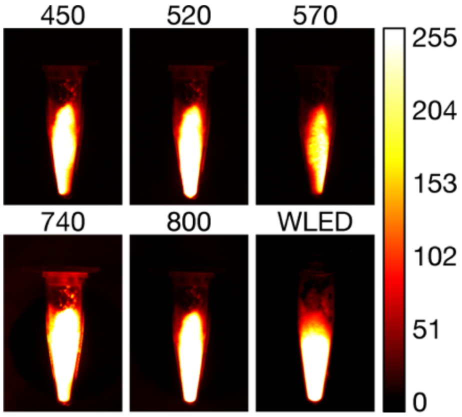

Supplementary Fig. 34 | NIR-imaging of Er-NCs exposed to single LED (exposure: 25 ms; filter: 1500LP).

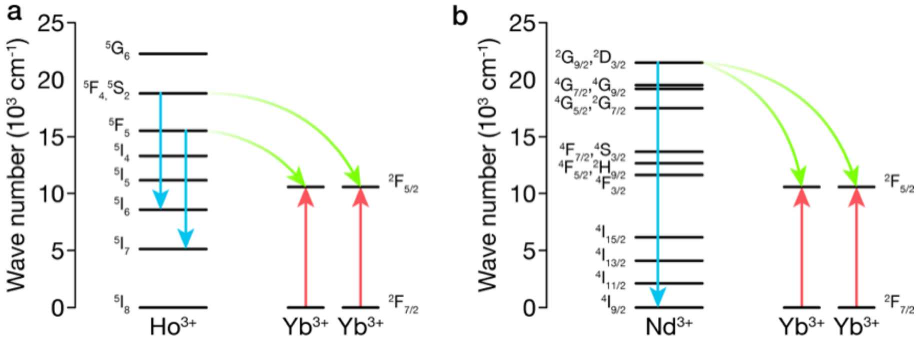

Supplementary Fig. 35 | Energy-level diagrams schematically illustrating the energy transfer mechanisms of two-photon NIR quantum cutting of **a**, Ho^{3+}/Yb^{3+} pair and **b**, Nd^{3+}/Yb^{3+} pair.

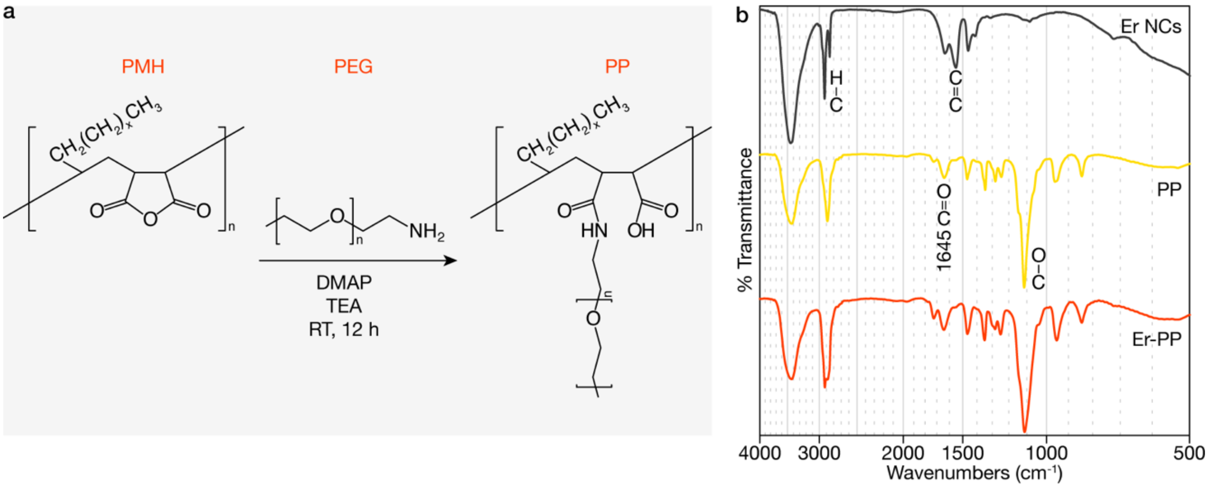

Supplementary Fig. 36 | a, Synthetic route of the amphiphilic polymer, poly(maleic anhydride-alt-1-octadecene)-PEG (PMH-PEG, PP). **b**, FTIR spectra of Er-NCs, PP, and Er-PP. For Er-PP sample. The existence of C-O bonds confirmed the successful modification of Er-NCs with PP.

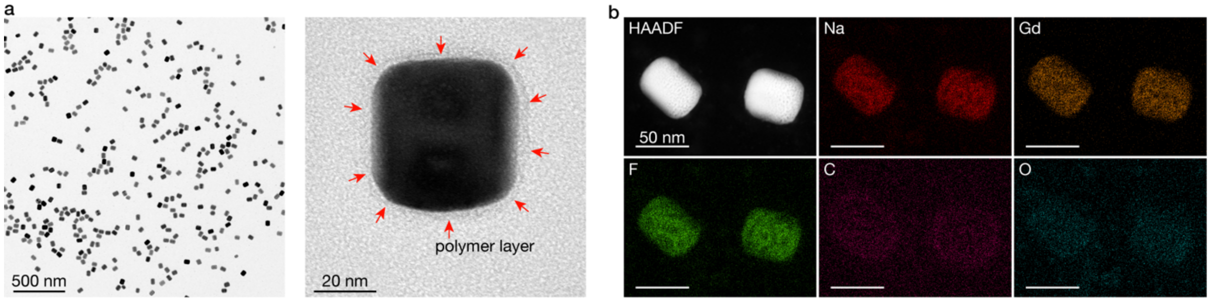

Supplementary Fig. 37 | a, TEM images of Er-PP NCs. **b**, STEM-EDS elemental mapping of Er-PP NCs.

HRTEM image displayed the existence of a thin polymer layer (~3.5 nm) upon the surface of Er-NCs. Elemental mapping confirmed the abundance of PP polymer by the signal of C and O.

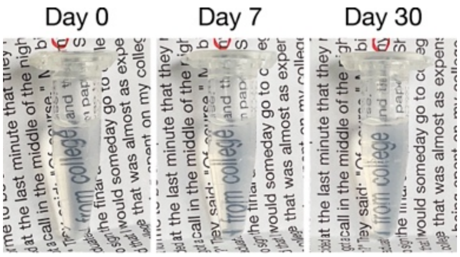

Supplementary Fig. 38 | Images of the normal saline solution of Er-PP NCs (1 mg mL^{-1}) stored at room temperature for various days.

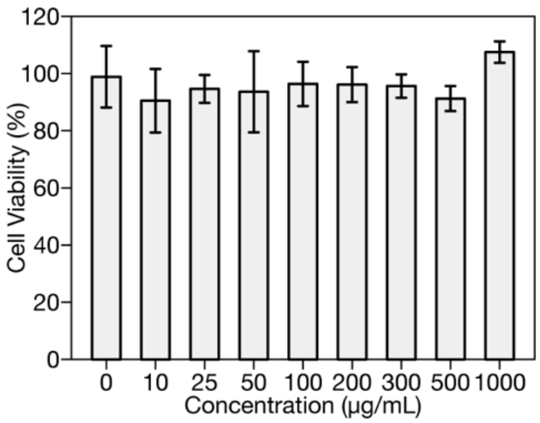

Supplementary Fig. 39 | Cytotoxicity of the as-prepared Er-PP probe, error bars in the figure represent standard deviation.

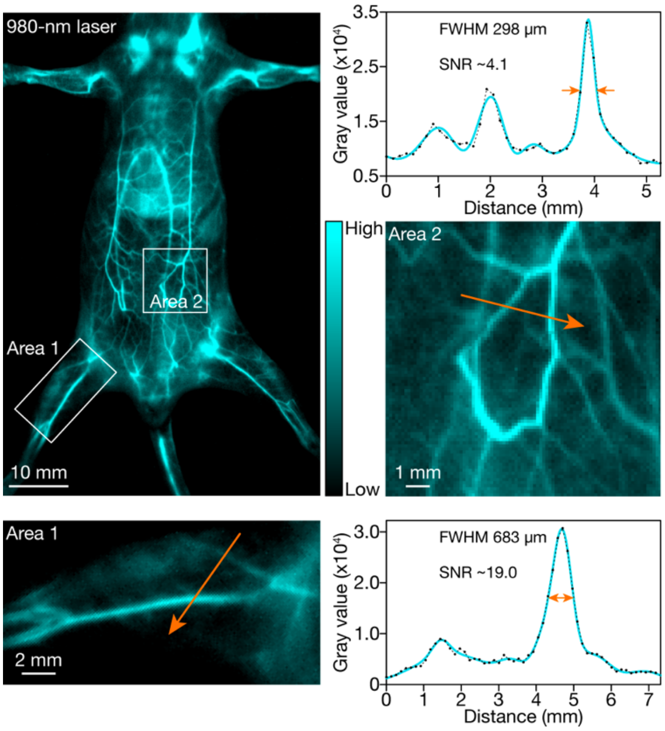

Supplementary Fig. 40 | Whole-body angiography of a mouse using a 980-nm laser as the excitation source, and the analysis on cross-sectional intensity (black line) and Gaussian fit fluorescence intensity profiles (cyan line).

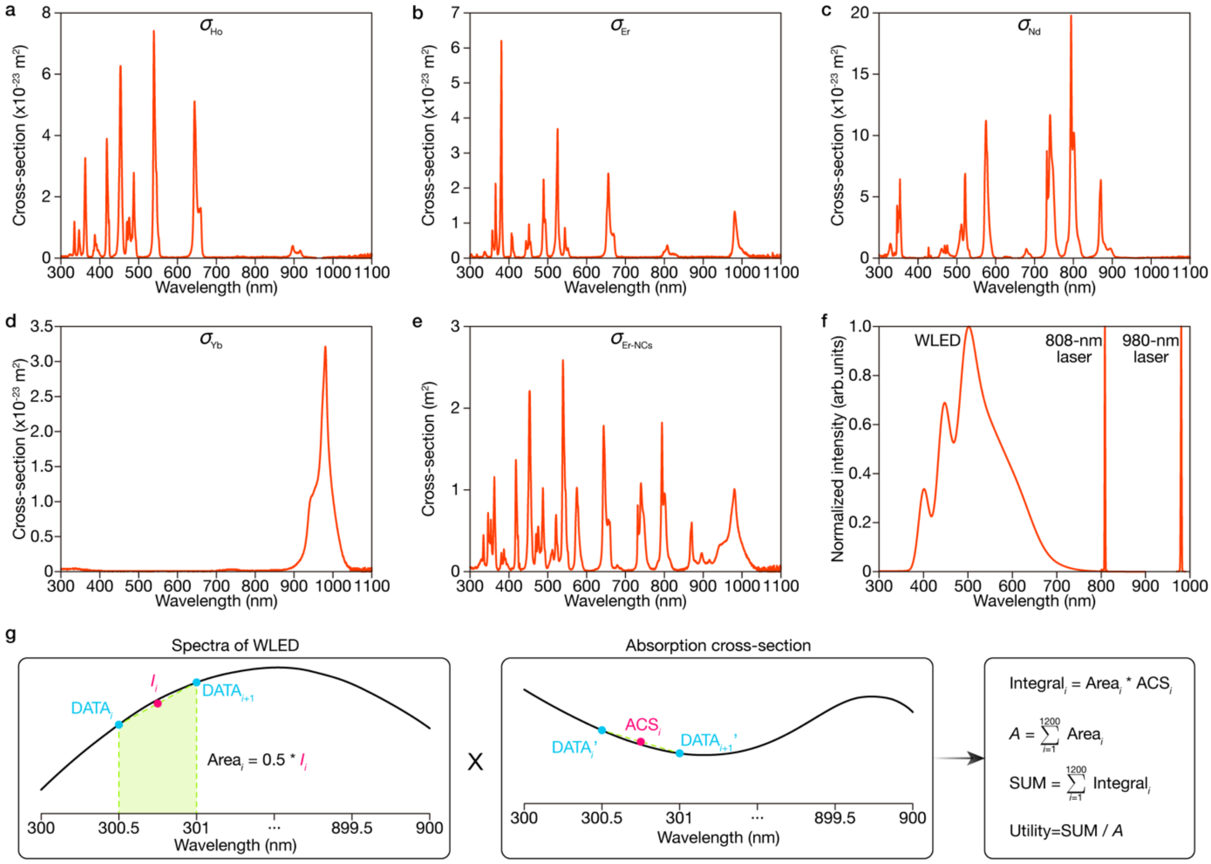

Supplementary Fig. 41 | a-d, Absorption cross-section of **a**, Ho^{3+}, **b**, Er^{3+}, **c**, Nd^{3+}, and **d**, Yb^{3+}. **e**, Calculated absorption cross-section of 1 mol of Er-NCs. **f**, Radiation spectra of the adopted 808-nm laser, 980-nm laser, and WLED. **g**, Principle for the calculation of absorptivity of Er-NCs to the specific light source.

The absorption cross-section of Er-NCs was calculated from the absorption cross-sections of Ho^{3+}, Er^{3+}, Nd^{3+}, and Yb^{3+}. The absorption spectra of $HoCl_3$, $ErCl_3$, $NdCl_3$, and $YbCl_3$ aqueous solution (c=0.2 M) were tested first. Let A be the absorbance at a specific wavelength, ε be the molar extinction coefficient, σ be the effective cross-section for absorption in m^2, and d be the thickness of the sample. According to the Beer-Lambert equation, we obtain

$$A = \varepsilon c d \tag{1}$$

Since N_A (Avogadro's number) = 6.022 x 10^{23}, we obtain

$$\sigma = \varepsilon / N_A = 1.66 \times 10^{-24}\ \varepsilon \tag{2}$$

So, the absorption cross-section of Ho^{3+}, Er^{3+}, Nd^{3+}, and Yb^{3+} could be calculated (Supplementary Fig. 41a-d).

The ICP test confirmed the mole fractions of Ho, Er, Nd, and Yb in Er-NCs were 5.78%, 0.54%, 1.51%, and 5.98%, respectively. Since the absorption of Er-NCs was mainly contributed by Ho, Er, Nd and Yb, the absorption cross-section of 1 mol of Er-NCs (Supplementary Fig. 41e) could be calculated by

$$\sigma = N_A\,(0.0578\ \sigma_{Ho} + 0.0054\ \sigma_{Er} + 0.0151\ \sigma_{Nd} + 0.0598\ \sigma_{Yb}) \tag{3}$$

To study the absorptivity of Er-NCs to 808-nm laser, 980-nm laser, and WLED, the radiation spectra of these three sources were measured first (Supplementary Fig. 41f). Then a numerical integration of

the spectra was performed using the absorption cross-section of Er-NCs as a weight. The integration was finally normalized to the area of the spectrum to characterize the absorptivity of Er-NCs to unit energy from the excitation sources. The principle for the calculation was shown in Supplementary Fig. 41g and the MATLAB codes were pasted below.

Finally, we obtained the absorptivity of Er-NCs to unit energy of 808-nm laser was 0.2687 m^2, to that of the 980-nm laser was 0.9867 m^2, and to that of WLED was 0.3515 m^2.

Despite the low absorption cross-section (Supplementary Fig. 41d), abundant Yb^{3+} in sensitizer-layers and activator-layer contribute to the high absorptivity of Er-NCs to 980-nm laser. Besides, 980-nm photons have higher penetration depth. These two factors together lead to the best angiography effect. Er-NCs even have a higher absorptivity to the energy from WLED than that from the 808-nm laser. But the energy conversion efficiency and the penetration capacity differ for these two types of sources, so the 808-nm laser pumped case still produces a better effect.

– –

Used MATLAB codes:

```
clear all
clc

DATA=xlsread('Spectra1.xlsx');              % data input
step=0.5;                                   % step of spectrum measurement
I=zeros(1200,1);
ACS=zeros(1200,1);
Area=zeros(1200,1);
Integral=zeros(1200,1);

for i=1:1:1200
   I(i,1)=0.5*(DATA(i,1)+DATA(i+1,1));       % the average value of DATA_i and DATA_i+1
   Area(i,1)=step*I(i,1);                    % spectrum area between interval of x_i and x_i+1
   ACS(i,1)=0.5*(DATA(i,2)+DATA(i+1,2));     % average value of absorption cross-section (σ)
   Integral(i,1)=step*I(i,1)*ACS(i,1);       % utilization efficiency between interval of x_i and x_i+1
end

A=sum(Area);
SUM=sum(Integral);
Utility=SUM/A;
```

– –

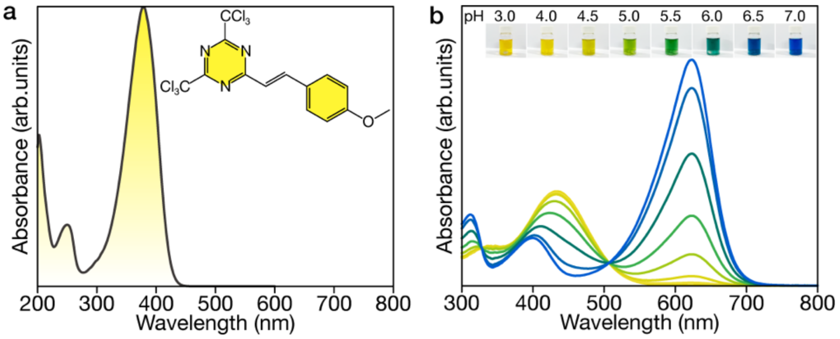

Supplementary Fig. 42 | a, Absorption spectrum of PAG. **b**, Absorption spectra and images of bromocresol green (BG) at different pH values.

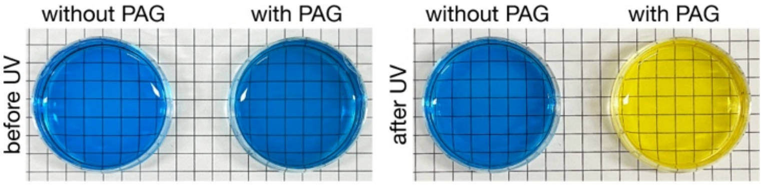

Supplementary Fig. 43 | Photochromism in ethanol solution. Only in the presence of PAG and exposure to UV light, can the solution change color.

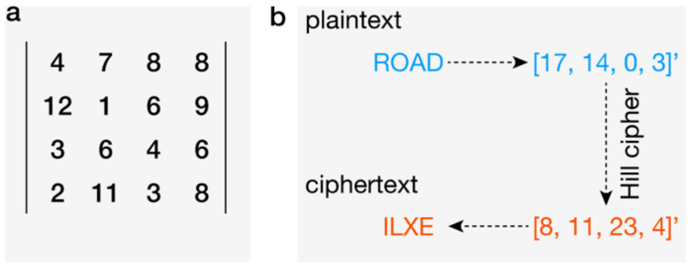

Supplementary Fig. 44 | a, Key matrix for Hill cipher. **b**, Hill cipher for the plaintext of "ROAD". Following the Hill encryption algorithm, the plaintext of "ROAD" could be switched into the ciphertext of "ILXE" by the self-defined key matrix.

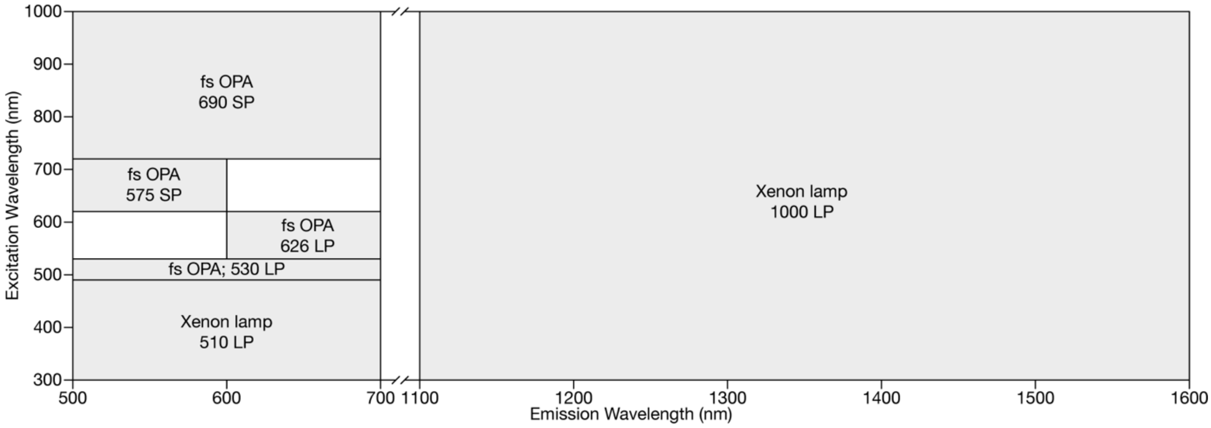

Supplementary Fig. 45 | The adopted configuration of excitation source and filter for the measurement of excitation-emission map.

Supplementary References

1. Mai, H.-X. et al. High-quality sodium rare-earth fluoride nanocrystals: controlled synthesis and optical properties. *J. Am. Chem. Soc.* **128**, 6426–6436 (2006).
