# Supplementary material for: Ultra-wideband-responsive photon conversion through co-sensitization in lanthanide nanocrystals
Source: Nat Commun. 2023 Feb 14;14:827. doi: 10.1038/s41467-023-36510-3 (PMC9929054; doi:10.1038/s41467-023-36510-3)
Supplement: Supplementary file 3 — Description to Additional Supplementary Information [file 41467_2023_36510_MOESM3_ESM.pdf]

Description of Additional Supplementary Files

Supplementary Video 1

NIR fluorescence imaging of Er-NCs sample sequentially pumped by multiple LEDs.

Supplementary Video 2

Whole-body angiography of a mouse using an 808-nm laser as the excitation source.

Supplementary Video 3

Whole-body angiography of a mouse using a white LED as the excitation source.

Supplementary Video 4

Whole-body angiography of a mouse using a 980-nm laser as the excitation source.

Supplementary Video 5

Photochromism of “BG (bromocresol green) + PAG (photo-acid generator)” system in ethanol solution and in PMMA film.
